# GPX4 restricts ferroptosis of NKp46^+^ILC3s to control intestinal inflammation

**DOI:** 10.1038/s41419-024-07060-3

**Published:** 2024-09-19

**Authors:** Xinyao Li, Junyu He, Xiang Gao, Guilang Zheng, Chunling Chen, Yimin Chen, Zhe Xing, Tianci Wang, Jian Tang, Yuxiong Guo, Yumei He

**Affiliations:** 1 Pediatric Intensive Care Unit, Guangdong Provincial People’s Hospital (Guangdong Academy of Medical Sciences); Department of Immunology, School of Basic Medical Sciences; Department of Clinical Laboratory, The Third Affiliated Hospital of Southern Medical University, Southern Medical University, Guangzhou, China; 2 Department of Immunology; Guangdong Provincial Key Laboratory of Single Cell Technology and Application, School of Basic Medical Sciences, Southern Medical University, Guangzhou, China; 3 Department of Gastroenterology, the Sixth Affiliated Hospital, Sun Yat-sen University, Guangzhou, China; 4 Pediatric Intensive Care Unit, Guangdong Provincial People’s Hospital (Guangdong Academy of Medical Sciences), Southern Medical University; Guangdong Provincial Cardiovascular Institute, Guangdong Provincial People’s Hospital, Guangdong Academy of Medical Sciences, Guangzhou, China

**Keywords:** Immune cell death, Innate lymphoid cells, Acute inflammation

## Abstract

Group 3 innate lymphoid cells (ILC3s) are essential for both pathogen defense and tissue homeostasis in the intestine. Dysfunction of ILC3s could lead to increased susceptibility to intestinal inflammation. However, the precise mechanisms governing the maintenance of intestinal ILC3s are yet to be fully elucidated. Here, we demonstrated that ferroptosis is vital for regulating the survival of intestinal ILC3. Ferroptosis-related genes, including GPX4, a key regulator of ferroptosis, were found to be upregulated in intestinal mucosal ILC3s from ulcerative colitis patients. Deletion of GPX4 resulted in a decrease in NKp46^+^ILC3 cell numbers, impaired production of IL-22 and IL-17A, and exacerbated intestinal inflammation in a T cell-independent manner. Our mechanistic studies revealed that GPX4-mediated ferroptosis in NKp46^+^ILC3 cells was regulated by the LCN2-p38-ATF4-xCT signaling pathway. Mice lacking LCN2 in ILC3s or administration of a p38 pathway inhibitor exhibited similar phenotypes of ILC3 and colitis to those observed in GPX4 conditional knock-out mice. These observations provide novel insights into therapeutic strategies for intestinal inflammation by modulating ILC3 ferroptosis.

## Introduction

Innate lymphoid cells (ILCs), a group of lymphocytes derived from common lymphoid progenitor cells, exhibit a similar morphology to adaptive lymphocytes and lack somatically recombined antigen-specific receptors but are characterized by surface CD127 (IL-7Rα) enrichment [1, 2]. According to transcription factor and cytokine expression patterns, ILCs are classified into three distinct groups, Group 1 (ILC1s), Group 2 (ILC2s), and Group 3 (ILC3s) [3]. ILC3s are characterized by retinoic acid-related orphan receptor γt (RORγt) expression and IL-22 and IL-17 production [4, 5]. ILC3s predominantly reside within the intestinal mucosal tissue and play a pivotal role in maintaining intestinal mucosal homeostasis and orchestrating inflammatory responses [6]. The composition of intestinal ILC3s comprises diverse heterogeneous subpopulations characterized by their distinct expression patterns of CCR6 and NKp46 [4, 7–9].

The role of ILC3s in regulating intestinal immunity is a double-edged sword. First, their activation triggers the production of antimicrobial peptides, such as RegIII β and RegIII γ, by epithelial cells through IL-22, IL-17, and GM-CSF secretion to protect the intestinal mucosa from various pathogens [10–12]. Notably, IL-22 derived from ILC3s is essential for maintaining the integrity of the intestinal epithelium, stimulating antimicrobial peptide production to eliminate pathogens, and preventing microbial dysbiosis [13]. Conversely, excessive secretion of IL-17 and IL-22 can trigger an exaggerated neutrophil response, exacerbating barrier damage and contributing to colitis [1]. The aberrant distribution and dysfunction of ILC3 subgroups are pivotal factors in the pathogenesis of intestinal inflammatory diseases [14, 15]. The balance of functional ILC3s regulates inflammatory bowel diseases (IBD), such as ulcerative colitis (UC) and Crohn’s disease [16, 17]. Repairing intestinal mucosal damage and restoring intestinal barrier function are considered therapeutic approaches for UC [18, 19]. Restoring the proportion of IL-22^+^ ILC3s in the intestinal lamina propria could ameliorate the pathological signs observed in mice with UC [20]. Evidence suggests that targeting the distribution and functional equilibrium of distinct ILC3 subpopulations provides novel insights into UC treatment. However, our current understanding of the critical molecules and specific molecular mechanisms involved in ILC3-mediated UC regulation remains inadequate.

Ferroptosis, a novel form of regulated cell death, is characterized primarily by iron and lipid reactive oxygen species (ROS) accumulation [21], accompanied by distinct morphological alterations, such as mitochondrial contraction, condensed dense mitochondrial membranes, and fewer mitochondrial cristae [22, 23]. Although initially discovered as iron-dependent cell death in cancer cells [22], ferroptosis has recently been confirmed to play a crucial role in the pathogenesis of various human diseases, including systemic lupus erythematosus and rheumatoid arthritis [24, 25]. Moreover, the effects of ferroptosis on both innate and adaptive immune cells, including neutrophils [26], macrophages [27], NK cells [28], and Treg cells [29] have also been elucidated. Additionally, accumulating evidence suggests a significant association between ferroptosis and UC. Research has identified ferroptosis in DSS-induced UC mouse models, which leads to an aberrant immune infiltration [30]. Ferroptosis inhibitors, such as iron chelators, can effectively reduce ROS levels in the colon tissues of patients with UC, improve clinical symptoms and endoscopic manifestations [31], and alleviate immune infiltration in UC-model mice [32]. Furthermore, activation of glutathione peroxidase 4 (GPX4) can significantly suppress ferroptosis and ameliorate IBD-associated symptoms [33]. This suggests that ferroptosis is a potential therapeutic target for UC [34–36]. Ferroptosis is negatively regulated by xCT (solute carrier family 7a member 11, SLC7A11), GPX4, and GSH [37]. Furthermore, several transcription factors, including ETS1, ATF4, and NRF2, control xCT expression [38–40]. Despite the recognition of xCT [41] and GPX4 [42] as key regulators in ferroptosis, the precise molecular mechanisms underlying their involvement in the progression of UC disease remain elusive. Moreover, the potential association between the regulatory role of ILC3s as a component of innate immunity and ferroptosis within the context of UC remains unknown.

Herein, we successfully demonstrated, for the first time, the role of ferroptosis of ILC3s in intestinal inflammation, providing novel insights into the potential role of immune cell ferroptosis in UC pathogenesis. Our findings will facilitate the development of novel therapeutic strategies against UC.

## Results

### Altered ferroptosis-regulated molecule expression patterns accompany ILC3 activation

**Fig. 1:**
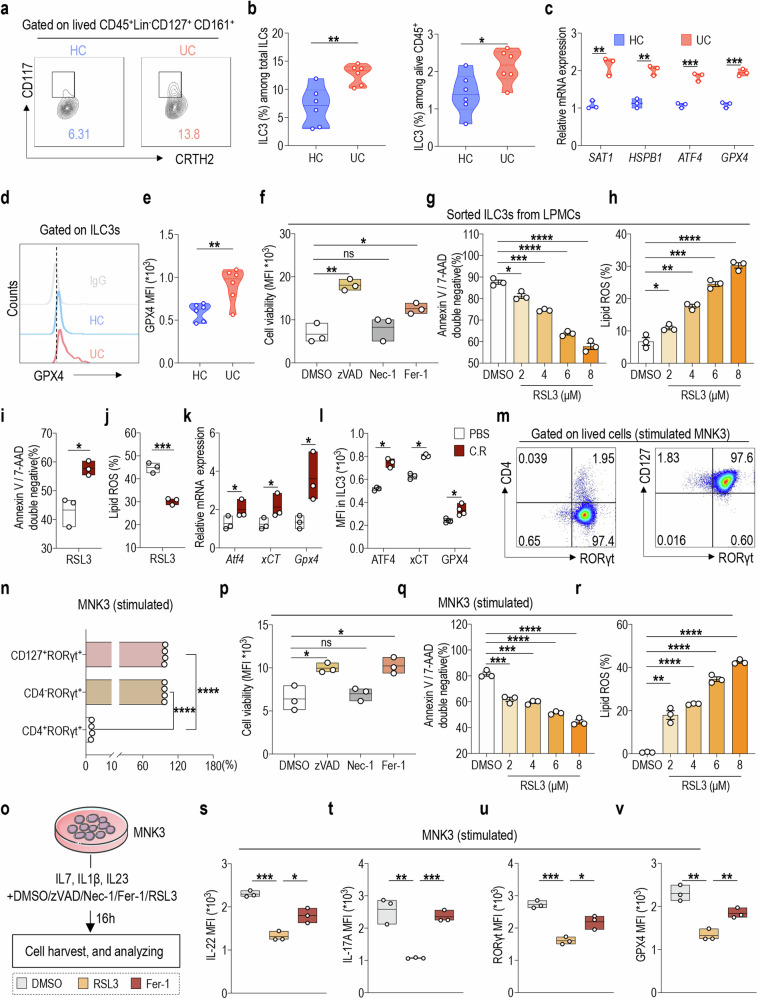
Altered ferroptosis-regulated molecule expression patterns accompany ILC3 activation. **a** The representative flow cytometry plots and **b** statistical results of ILC3 proportion in total ILCs (left) and in CD45^+^ cells (right) in intestinal mucosal tissues of UC patients compared with those in HCs. (*n* = 6) **c** *SAT1*, *HSPB1*, *ATF4* and *Gpx4* mRNA expression in the sorted ILC3s from intestinal mucosal tissue of HCs or patients with UC. Relative gene expression was normalized to β-actin. (*n* = 3) **d, e** Comparison of GPX4 expression in ILC3s from indicated intestinal mucosal tissue. (*n* = 6) **f** Sorted ILC3s from the LPMCs were cultured with the indicated inhibitors for 24 h: zVAD (10 μM), necrostatin-1 (Nec-1, 1 μM) and ferrostatin-1 (Fer-1, 1 μM). Cell viability was measured using the alamarBlue cell viability assay. (*n* = 3) **g**, **h** Sorted ILC3s from LPMCs were treated with RSL3 (2–8 μM) or vehicle (DMSO) for 16 h. **g** The percentage of 7-AAD and annexin V double-negative cells was used to gauge cell viability, as described previously [74, 75]. **h** Lipid ROS production was assayed by flow cytometry using C11-BODIPY. The statistical analysis is shown. (*n* = 3) WT mice were administered with or without (PBS) 1 × 10^8^ CFU of *C. rodentium* (C.R) via oral gavage. After 8 days, mice were euthanized, and their intestinal tissue was collected. Sorted intestinal LPMC-derived ILC3s from the indicated mice were treated with RSL3 (8 μM) for 16 h. Statistical results of **i** the percentage of 7-AAD and annexin V double-negative population, and **j** lipid ROS production, are shown. (*n* = 3) **k** *Atf4*, *xCT*, and *Gpx4* mRNA expression in the sorted intestinal ILC3s from the indicated mice was assayed using qRT-PCR. The relative gene expression was normalized to β-actin. (*n* = 3) **l** The statistical results of MFI for ATF4, xCT, and GPX4 in ILC3s from the indicated mice are presented. (*n* = 3) **m** MNK3 cells were stimulated with IL-7, IL1β, and IL-23 (10 ng/mL) in vitro for 24 h. The viable MNK3 cells were gated on live cells and further analyzed for CD4, CD127, and RORγt expression using flow cytometry. **n** The statistical results depicting the proportions of CD127^+^RORγt^+^, CD4^-^RORγt^+^, and CD4^+^RORγt^+^ cell populations are shown. (*n* = 4) **o** In the presence of IL-7, IL1β, and IL-23 (10 ng/mL) for MNK3 cell stimulation, the specified inhibitors or inducer were applied to treat MNK3 cells: zVAD (10 μM), Nec-1 (Nec-1, 1 μM), ferrostatin-1 (Fer-1, 1 μM) or RSL3. After 16 h of cultivation, cells were harvested for analysis. **p** Cell viability was measured using the alamarBlue cell viability assay. (*n* = 3) Flow cytometry results of **q** the percentage of 7-AAD and annexin V double-negative cells, and **r** lipid ROS production, are shown. (*n* = 3) The statistical results of the MFI for **s** IL-22, **t** IL-17A, **u** RORγt, and **v** GPX4 in activated MNK3 cells treated with RSL3 or Fer-1 are presented. (*n* = 3) Data are presented as the mean ± SEM or median, and statistical significance was determined by two-sided unpaired *t*-test (**b**, **c**, **e**–**j**, **l**, **n**, **p**–**v**) or non-parametric Mann–Whitney *U* test (K). *P < 0.05; **P < 0.01; ***P < 0.001.

The association between ferroptosis and UC has been extensively documented; however, the potential regulatory mechanism of ILC3s in UC with respect to its relationship with ferroptosis remains elusive. To address this, we collected intestinal mucosal tissue from UC patients for further investigation. We observed a significant increase in the proportion of ILC3s derived from intestinal mucosal tissue in UC patients compared to that in healthy controls (HC) (Fig. 1a, b). Furthermore, several ferroptosis-related genes were upregulated in ILC3s derived from intestinal tissue of UC patients, including *SAT1, HSPB1, ATF4*, and *GPX4* (Fig. 1c). These findings are consistent with the results obtained through reanalysis of single-cell sequencing data previously reported by Smillie et al. in patients with UC (2019, accession number SCP259) (Supplementary Fig. 1a) [43]. Notably, GPX4 expression was significantly enhanced in ILC3s of the intestinal mucosal tissue from patients with UC (Fig. 1d, e). Therefore, this phenomenon suggests that the ferroptosis-associated molecular mechanisms may be involved in the ILC3-mediated regulation of intestinal inflammation.

To further elucidate the contribution of ferroptosis to ILC3 death, ILC3s were isolated from the lamina propria mononuclear cells (LPMCs) of wild-type (WT) mice (Supplementary Fig. 1b) and cultured for 24 h with an apoptosis inhibitor (z-VAD-FMK, zVAD), necrosis inhibitor (Nec-1), or specific inhibitor of ferroptosis (ferrostatin-1, Fer-1) [44, 45]. The results demonstrated that zVAD and Fer-1 significantly enhanced the viability of LPMC-derived ILC3s, whereas Nec-1 did not affect cell survival (Fig. 1f). To evaluate the inherent susceptibility of ILC3s derived from LPMCs to ferroptosis, these cells were cultured in vitro with RSL3, a known ferroptosis inducer. The findings showed a decline in cellular viability (Supplementary Fig. 1c, Fig. 1g) and an accumulation of intracellular lipid ROS (Supplementary Fig. 1d, Fig. 1h) in a concentration-dependent manner after RSL3 treatment. These results suggest that ferroptosis contributes to the death of ILC3s and that ILC3s in intestinal tissue exhibit inherent susceptibility to ferroptosis.

Ferroptosis-regulated molecule expression patterns were further explored in ILC3s during intestinal inflammation. Infection with *C. rodentium* (C.R) induces disruption of the intestinal barrier, infiltration of neutrophils, and secretion of pro-inflammatory cytokines in mice, thereby establishing the mouse model as an ideal representation of human UC [46, 47]. Therefore, the C.R intestinal infection model was employed (Supplementary Fig. 2a). The findings demonstrated that sorted LPMC-derived ILC3s showed an increase in cell viability and a decrease in intracellular lipid ROS in a concentration-dependent manner following treatment with RSL3 after C.R infection (Supplementary Fig. 2b, c, Fig. 1i, j), indicating reduced sensitivity of activated ILC3s to RSL3 treatment. However, these effects were reversed upon administration of Fer-1. Consistent with the data obtained from human UC, *Sat1, Hspb1, Atf4, xCT*, and *Gpx4* mRNA expression was significantly upregulated in sorted LPMCs-derived ILC3s after C.R infection. In contrast, the expression of *Nrf2* and *Ets1*, which control xCT expression, did not change significantly (Fig. 1k, Supplementary Fig. 2d). ATF4, XCT, and GPX4 protein expression was significantly increased (Supplementary Fig. 2e, Fig. 1l), suggesting a shift toward an anti-ferroptotic expression pattern with intestinal inflammation. These results further support the involvement of ferroptosis molecular mechanisms in the ILC3-mediated regulation of intestinal inflammation.

To verify these conclusions, the ILC3 cell line MNK3 [48], exhibiting RORγt and CD127 expression, while lacking CD4 expression (Fig. 1m, n), was cultured in vitro with the addition of inhibitors or a ferroptosis inducer and cytokine stimulation (Supplementary Fig. 3a, Fig. 1o). Consistent with observations in the mouse C.R infection model, ferroptosis contributed to MNK3 cell death, and the activated MNK3 cells showed intrinsic susceptibility to ferroptosis (Supplementary Fig. 3b, c, and Fig. 1p–r). Further, the sensitivity of activated MNK3 cells to RSL3 treatment was diminished compared with that in the resting state, and this effect was reversed upon Fer-1 administration (Supplementary Fig. 3b, c). Meanwhile, after MNK3 cell activation, *Atf4, xCT*, and *Gpx4* mRNA and protein expression was significantly upregulated (Supplementary Fig. 3d–f). Notably, after RSL3 treatment, IL-22 and IL-17A cytokine secretion by activated MNK3 cells was impaired, but this was recovered upon Fer-1 administration, accompanied by the restoration of RORγt and GPX4 expression (Fig. 1s–v). Together, these data reveal that the potential molecular mechanism underlying ferroptosis involves the regulation of intestinal inflammation by ILC3s, specifically through the modulation of ILC3 survival activity and secretion of IL-22/IL-17A.

### NKp46^+^ILC3 orchestrates pathological phenotypic alterations in mouse colitis induced by in vivo ferroptosis interventions, independent of T cell involvement

**Fig. 2:**
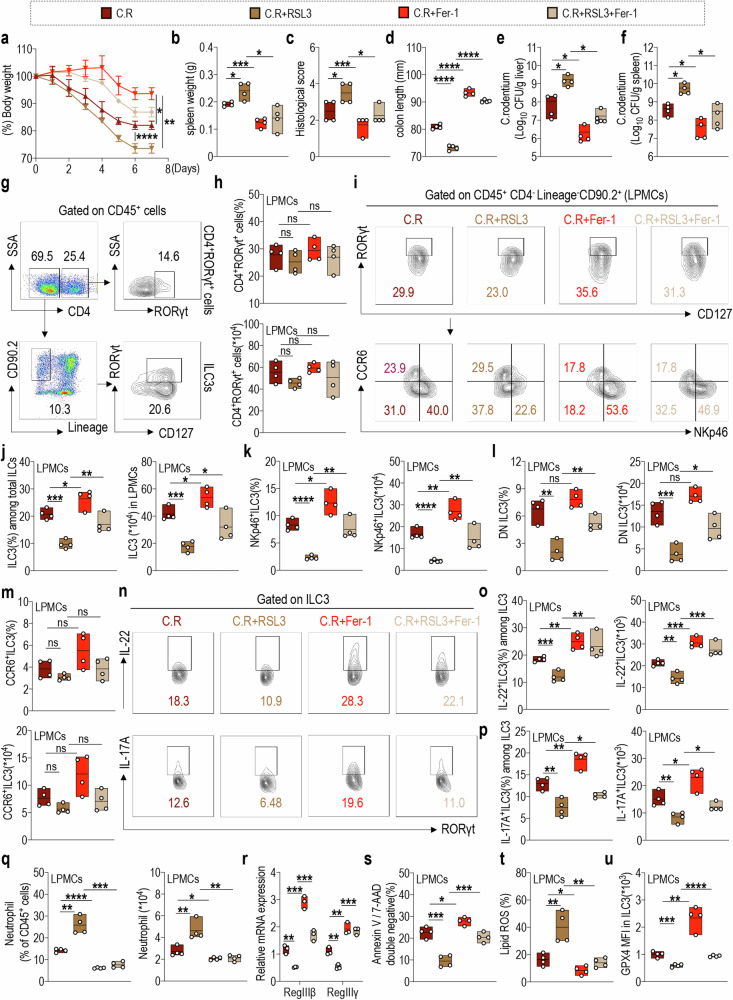
NKp46^+^ILC3 orchestrates pathological phenotypic alterations in mouse colitis induced by in vivo ferroptosis interventions, independent of T cell involvement. **a** Body weight changes of indicated groups of mice. (*n* = 3) **b** Statistical results of spleen weights at day 8 post infection. (*n* = 4) **c** The statistical results of histological scores are shown. (*n* = 4) **d** Colon lengths of indicated mice. (*n* = 4) Log_10_ CFU of C.R in **e** liver and **f** spleen tissues. (*n* = 4) **g** The gating strategy for intestinal CD4^+^RORγt^+^ cells and ILC3s. **h** Statistical results of the proportion (upper) and absolute number (lower) of CD4^+^RORγt^+^ cells in LPMCs from the indicated groups of mice. (*n* = 4) **i** The representative flow cytometry plots and statistical results of the proportion and absolute number of **j** ILC3s, including **k** NKP46^+^ILC3, **l** DN cell subsets, and **m** CCR6^+^ILC3 in LPMCs of indicated mice. (*n* = 4) **n** Representative FACS plots of IL-22- (upper) and IL-17A-positive (lower) ILC3s in LPMCs from the indicated groups of mice. Statistical results of proportion (left) and absolute numbers (right) of **o** IL-22-, and **p** IL-17A-expressing ILC3s are shown. (*n* = 4) **q** Statistical results of the proportion (left) and numbers (right) of neutrophils in LPMCs from the indicated groups of mice. (*n* = 4) **r** Relative RegIIIβ and RegIIIγ mRNA expression in the colon tissue of indicated mice. (*n* = 3) **s** The percentage of annexin V and 7-AAD double-negative population in LPMCs-derived ILC3s from the indicated groups of mice. (*n* = 4) **t** Statistical results of lipid ROS production in ILC3s are shown. (*n* = 4) **u** The statistical results of MFI for GPX4 in ILC3s from LPMCs of indicated mice. (*n* = 4) Data are presented as the mean ± SEM or median, and statistical significance was determined by one-way ANOVA test (**b**–**f, h, j**–**m, o**–**u**). *P < 0.05; **P < 0.01; ***P < 0.001.

A C.R infection mouse model was used to investigate the specific role of ferroptosis in the regulation of intestinal inflammation. Mice were treated with RSL3, Fer-1, or both (Supplementary Fig. 4a). After C.R infection, mice exhibited significant weight loss from day 4 (Fig. 2a) and an increase in spleen weight (Fig. 2b, Supplementary Fig. 4b). H&E staining revealed that colonic pathology, characterized by immune cell infiltration, crypt proliferation, and edema, was augmented (Fig. 2c, Supplementary Fig. 4c). Additionally, a reduction in colon length was observed (Fig. 2d, Supplementary Fig. 4d), along with an increased bacterial load in the mouse liver and spleen following C.R infection (Fig. 2e, f). These pathological alterations were exacerbated upon treatment with RSL3 but ameliorated when treated with Fer-1. Importantly, Fer-1 administration reversed the exacerbated pathological changes induced by RSL3.

Subsequently, we elucidated the specific coordinated immune cell populations involved in the pathological alterations induced by intervention in ferroptosis. Considering the pivotal role of RORγt^+^ cells in orchestrating immune responses, inflammation, and tolerance within the intestinal environment [49–51], we initially assessed intestinal CD4^+^RORγt^+^ cell proportions. The results revealed no significant alterations in CD4^+^RORγt^+^ cell proportions and numbers in C.R-infected mice treated with RSL3 or Fer-1 (Fig. 2g, h). However, following C.R infection, a significant increase in the proportion and absolute number of ILC3s (CD4^−^CD127^+^RORγt^+^ cells) (Fig. 2i, j) from mesenteric lymph nodes (mLNs) (Supplementary Fig. 4e, f) and LPMCs was observed. This increase was primarily evident in the NKp46^+^ILC3 (Fig. 2k) and DN ILC3 (Fig. 2l) cell subsets, with particularly pronounced changes seen in NKp46^+^ILC3s, which exhibited a notable decrease following RSL3 administration; however, their levels were restored upon treatment with Fer-1. Meanwhile, no notable changes were detected in CCR6^+^ILC3s (Fig. 2m). RSL3 treatment significantly decreased the proportion and absolute number of IL-22^+^ILC3s and IL-17A^+^ILC3s among LPMCs from mice after C.R infection. However, this effect was markedly reversed upon Fer-1 treatment (Fig. 2n-p). We observed a significant recruitment of intestinal neutrophils in the C.R-infected mice after RSL3 treatment, both in proportion and number (Fig. 2q). Moreover, the release of the antimicrobial peptides RegIIIβ and RegIIIγ by intestinal epithelial cells (IECs) was reduced (Fig. 2r), indicating an exacerbated barrier damage; however, Fer-1 administration effectively reversed these effects. Additionally, RSL3 treatment exacerbated ferroptosis characteristics of intestinal ILC3s, which included a reduction in cell viability (Fig. 2s), an increase in intracellular lipid ROS accumulation (Fig. 2t), and a decrease in GPX4 expression (Fig. 2u). However, these effects were significantly reversed upon Fer-1 treatment, accompanied by the restoration of GPX4 expression in the intestinal ILC3s from mice (Fig. 2u). Conversely, no significant alteration in GPX4 expression was detected in CD4^+^RORγt^+^ cells (Supplementary Fig. 4g). These results strongly suggest the involvement of ferroptosis in the regulation of intestinal inflammation in mice, primarily mediated by NKp46^+^ILC3s, thereby highlighting GPX4 as a potential pivotal regulatory molecule.

### NKp46^+^ILC3 regulation in colitis requires GPX4 for pathogen resistance and barrier repair

To better understand the specific role of GPX4 in ILC3, Gpx4^fl/fl^Rorc^cre^ mice were employed to delete GPX4 in RORγt-positive cells. We initially investigated the potential regulatory role of GPX4 in ILC3s under steady-state conditions and revealed no significant alterations in intestinal length (Supplementary Fig. 5a, b), spleen weight (Supplementary Fig. 5c, d), and proportion and absolute number of ILC1s, ILC2s, and ILC3s in mLN and LPMCs from Gpx4^fl/fl^Rorc^cre^ mice compared with those from Gpx4^fl/fl^ mice (Supplementary Fig. 5e–j). Furthermore, we observed no notable changes in ferroptosis-associated characteristics in intestinal ILC3s from Gpx4^fl/fl^Rorc^cre^ mice, including cell viability (Supplementary Fig. 5k), intracellular lipid ROS accumulation (Supplementary Fig. 5l), and Fe^2+^ content (Supplementary Fig. 5m). We postulated the involvement of GPX4 in the regulation of ILC3-mediated acute colitis, as evidenced by its upregulated expression in ILC3s within the intestinal mucosa of patients with UC.

**Fig. 3:**
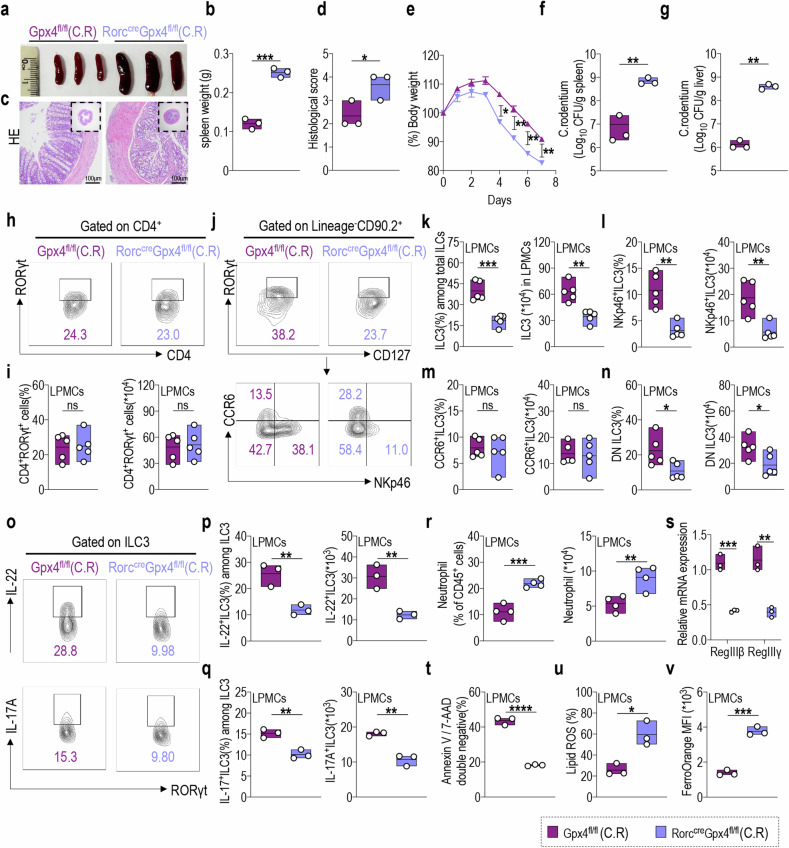
NKp46^+^ILC3 regulation in colitis requires GPX4 for pathogen resistance and barrier repair. Gpx4^fl/fl^ and Rorc^cre^Gpx4^fl/fl^ mice were orally inoculated with 1×10^8^ CFU of C.R, and their tissues were collected on Day 8 post-infection. **a** Representative spleen images and **b** statistical results of spleen weights at Day 8 post-infection. (*n* = 3) **c** Representative H&E staining of the colon sections from indicated mice with C.R infection (scale bars: 100 μm). **d** The statistical results of histological scores are shown. (*n* = 4) **e** Body weight changes of indicated mice. (*n* = 4) Log_10_ CFU of C.R in **f** liver and **g** spleen tissues on day 8 post infection. (*n* = 3) **h** The representative flow cytometry plots of CD4^+^RORγt^+^ cells in LPMCs from the indicated mice. **i** Statistical results of proportion (left) and absolute numbers (right) of CD4^+^RORγt^+^ cells are shown. (*n* = 4) **j** Representative flow cytometry plots and statistical results of the proportion (left) and absolute number (right) of **k** ILC3s, including **l** NKp46^+^ILC3, **m** CCR6^+^ILC3, and **n** DN cell subsets in LPMCs of Gpx4^fl/fl^ and Rorc^cre^Gpx4^fl/fl^ mice following C.R infection. (*n* = 4) **o** Representative FACS plots of IL-22- (upper) and IL-17A-positive (lower) ILC3s in LPMCs from the indicated mice. Statistical results of proportion (left) and absolute numbers (right) of **p** IL-22-, and **q** IL-17A-expressing ILC3s are shown. (*n* = 3) **r** Statistical results of the proportion (left) and numbers (right) of neutrophils in LPMCs from the indicated mice. (*n* = 4) **s** Relative RegIIIβ and RegIIIγ mRNA expression in the colon tissue of indicated mice. (*n* = 3) Statistical results of **t** the percentage of annexin V and 7-AAD double-negative population and **u** lipid ROS production in LPMC-derived ILC3s from the indicated mice. (*n* = 3) **v** Fe^2+^ level in ILC3s from LPMCs was analyzed using FerroOrange, and the statistical results are shown. (*n* = 3) Data are presented as the mean ± SEM or median, and statistical significance was determined using two-sided unpaired *t*-test (**b**, **d**–**g**, **i**, **k**–**n**, **p**–**v**). *P < 0.05; **P < 0.01; ***P < 0.001.

To test this hypothesis, we utilized a C.R infection mouse model known to activate ILC3 and induce colitis prior to T cell activation [52]. Severe intestinal inflammation was observed in Gpx4^fl/fl^Rorc^cre^ mice, characterized by a significant increase in spleen weight (Fig. 3a, b) and colon pathological score (Fig. 3c, d), and a significant decrease in body weight from day 4 post C.R infection (Fig. 3e). Gpx4^fl/fl^Rorc^cre^ mice exhibited higher bacterial load in their liver and spleen (Fig. 3f, g). Compared with those in Gpx4^fl/fl^ mice, the proportion and absolute number of CD4^+^RORγt^+^ cells in the LPMCs were not significantly altered in Gpx4^fl/fl^Rorc^cre^ mice after C.R infection (Fig. 3h, i). However, notable reductions in both the proportion and absolute number of ILC3s from mLNs (Supplementary Fig. 6a, b) and LPMCs (Fig. 3j, k) were observed in Gpx4^fl/fl^Rorc^cre^ mice, suggesting an independent regulatory effect of GPX4 on ILC3s, distinct from that in T cells. In line with the observed phenomenon following RSL3 treatment of WT mice subjected to C.R infection, compared with Gpx4^fl/fl^ mice, Gpx4^fl/fl^Rorc^cre^ mice exhibited a significant reduction in NKp46^+^ILC3 and DN ILC3 subsets following C.R infection, with a particularly pronounced decrease in the NKp46^+^ILC3 subset. Furthermore, CCR6^+^ILC3s remained unchanged (Fig. 3l–n). Importantly, the proportion and absolute number of IL-22^+^ILC3s and IL-17A^+^ILC3s in LPMCs from Gpx4^fl/fl^Rorc^cre^ mice were significantly diminished (Fig. 3o–q). Correspondingly, intestinal neutrophil proportion and number in Gpx4^fl/fl^Rorc^cre^ mice were significantly increased (Fig. 3r), along with a notable reduction in the secretion of the antimicrobial peptides RegIIIβ and RegIIIγ by IECs (Fig. 3s), indicating aggravated intestinal inflammation. Furthermore, intestinal ILC3s from Gpx4^fl/fl^Rorc^cre^ mice exhibited enhanced ferroptosis characteristics, including notably decreased cell viability (Fig. 3t), elevated intracellular lipid ROS accumulation (Fig. 3u), and increased Fe^2+^ contents (Fig. 3v). The MAPK signaling pathways are required for IL-23-mediated IL-22 production by ILC3s [53]. Next, p38 (Thr180/Tyr182) and ERK1/2 (Thr204/Thr187) phosphorylation levels were assessed. After C.R infection, no significant alterations in p38 and ERK1/2 phosphorylation levels in ILC3s of LPMCs from Gpx4^fl/fl^Rorc^cre^ mice were observed, compared with those in Gpx4^fl/fl^ mice. Furthermore, no significant changes in protein expression of the transcription factor ATF4 and its downstream target xCT were observed in ILC3s (Supplementary Fig. 6c). These data suggest that GPX4 specifically regulates ILC3s independently of T-cell regulation by modulating the function and ferroptosis characteristics of ILC3s in colitis to enhance pathogen resistance and barrier repair. Moreover, MAPK signaling, ATF4, and xCT may act upstream of GPX4.

### LCN2 specifically mediates NKp46^+^ILC3 ferroptosis susceptibility and functions in colitis through GPX4

**Fig. 4:**
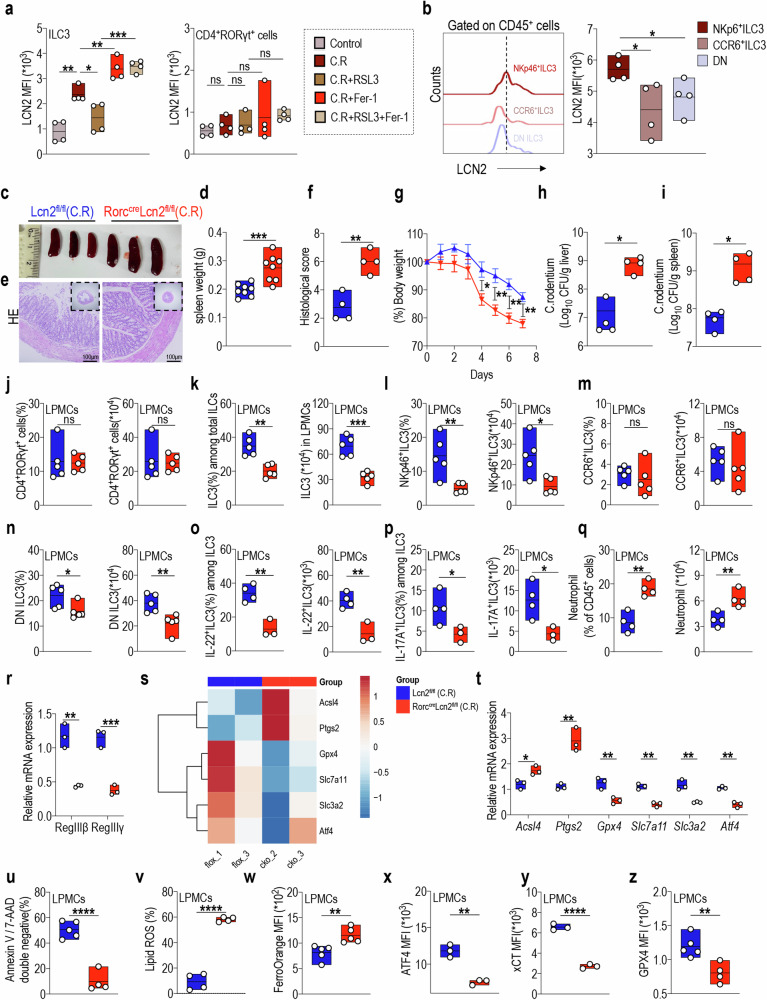
LCN2 specifically mediates NKp46^+^ILC3 ferroptosis susceptibility and functions in colitis through GPX4. **a** Statistical results of MFI for LCN2 expression in LPMC-derived ILC3s (left) and CD4^+^RORγt^+^ cells (right) after in vivo ferroptosis intervention in mice infected with C.R. (*n* = 4) **b** Comparison of LCN2 expression in NKp46^+^ILC3, CCR6^+^ILC3, and DN cell subsets from LPMCs of WT mice after C.R infection. (*n* = 4) Lcn2^fl/fl^ and Rorc^cre^Lcn2^fl/fl^ mice were orally administered with 1 × 10^8^ CFU of C.R, and their tissues were collected on Day 8 post-infection. **c** Representative spleen images and **d** statistical results of spleen weight of Lcn2^fl/fl^ and Rorc^cre^Lcn2^fl/fl^ mice on day 8 post infection. (*n* = 4) **e** Representative H&E-stained colon sections from indicated mice with C.R infection (scale bars: 100 μm). **f** The statistical results of histological scores are shown. (*n* = 4) **g** Body weight changes of indicated mice. (*n* = 4) Log10 CFU of C.R in **h** liver and **i** spleen tissues from indicated mice on day 8 post infection. (*n* = 4) **j** The comparison of CD4^+^RORγt^+^ cell proportions (left) and absolute numbers (right) in LPMCs of Lcn2^fl/fl^ and Rorc^cre^Lcn2^fl/fl^ mice post C.R infection. (*n* = 4) Statistical results of the proportion (left) and absolute number (right) of **k** ILC3s, including **l** NKp46^+^ILC3, **m** CCR6^+^ILC3, and **n** DN cell populations in LPMCs of the indicated mice after C.R infection. (*n* = 4) Statistical results of proportion (left) and numbers (right) of **o** IL-22-, and **p** IL-17A-positive ILC3s from LPMCs of indicated mice. (*n* = 4) **q** Statistical results of the proportion (left) and numbers (right) of neutrophils in LPMCs from the indicated mice. (*n* = 4) **r** Relative RegIIIβ and RegIIIγ mRNA expression in the colon tissue of the indicated mice. (*n* = 3) **s** Heatmap of differentially expressed genes related to ferroptosis in ILC3s from Lcn2^fl/fl^ and Rorc^cre^Lcn2^fl/fl^ mice following C.R infection. **t** Relative expression of mRNA related to ferroptosis in ILC3s isolated from LPMCs of indicated mice. (*n* = 3) Statistical results of **u** the percentage of annexin V and 7-AAD double-negative population and **v** lipid ROS production in LPMC-derived ILC3s from the indicated mice. (*n* = 4) **w** Fe^2+^ level in ILC3s from LPMCs was analyzed using FerroOrange, and the statistical results are shown. (*n* = 4) Statistical results of **x** ATF4, **y** xCT, and **z** GPX4 expression in ILC3s from LPMCs of indicated mice. (*n* = 4) Data are presented as the mean ± SEM or median, and statistical significance was determined using two-sided unpaired *t*-test (**a**, **b**, **d**, **f**–**r**, **t**–**z**). *P < 0.05; **P < 0.01; ***P < 0.001.

Lipocalin-2 (LCN2), also referred to as 24p3 or neutrophil gelatinase-associated lipocalin NGAL is a secreted glycoprotein in the lipocalin superfamily [54, 55]. The crucial role of LCN2 in iron homeostasis and inflammation has been well-demonstrated [56]. Importantly, LCN2 serves as a negative regulator of ferroptosis, thereby exerting control over the development of liver tumors [21]. Furthermore, increased expression and activation of LCN2 have been observed in patients with UC, suggesting its potential regulatory effect on colitis by mediating ferroptosis [57]. We therefore investigated the expression of LCN2 in ILC3s and elucidated its involvement in the complex regulatory mechanisms. In vivo intervention with ferroptosis consistently resulted in a pattern of changes in LCN2 expression similar to GPX4, characterized by inhibition of LCN2 expression following RSL3 treatment and subsequent recovery upon Fer-1 treatment (Fig. 4a). Conversely, no significant alteration was observed in LCN2 expression within CD4^+^RORγt^+^ cells (Fig. 4a). More than that, LCN2 expression was markedly higher in the NKp46^+^ILC3 than CCR6^+^ILC3 and DN ILC3 cell subpopulations (Fig. 4b), suggesting that LCN2 plays a crucial role in regulating ILC3s, particularly within the NKp46^+^ILC3 subset, through mechanisms independent of T cell regulation.

To further clarify the specific regulatory mechanism of LCN2 in ILC3s, Lcn2^fl/fl^Rorc^cre^ mice were used. First, we assessed LCN2 expression in ILC3s from LPMCs of Lcn2^fl/fl^Rorc^cre^ mice (Supplementary Fig. 7a). The results demonstrated that LCN2 expression in ILC3s from Lcn2^fl/fl^Rorc^cre^ mice was negligible compared with that in Lcn2^fl/fl^ mice, validating the efficacy of LCN2-knockout specifically in ILC3 cells (Supplementary Fig. 7b). Next, we examined the regulatory role of LCN2 in ILC3s under steady-state conditions. Consistent with findings in Gpx4^fl/fl^Rorc^cre^ mice, we observed no significant alterations in intestinal length (Supplementary Fig. 7c, d), spleen weight (Supplementary Fig. 7e, f), or proportions and absolute frequencies of ILC1s, ILC2s, and ILC3s in mLN and LPMCs from Lcn2^fl/fl^Rorc^cre^ mice (Supplementary Fig. 7g-l). Furthermore, no notable changes in ferroptosis-related characteristics in ILC3s of Lcn2^fl/fl^Rorc^cre^ mice, including cell viability (Supplementary Fig. 7m), intracellular lipid ROS accumulation (Supplementary Fig. 7n), and Fe^2+^ content, were observed (Supplementary Fig. 7o).

A C.R infection mouse model was applied to validate the LCN2-specific regulatory function in ILC3s. Lcn2^fl/fl^Rorc^cre^ mice displayed more severe intestinal inflammation, characterized by significant splenomegaly (Fig. 4c, d), elevated colonic pathological scores (Fig. 4e, f), and substantial weight loss starting from day 4 post C.R infection (Fig. 4g), consistent with observations in Gpx4^fl/fl^Rorc^cre^ mice infected with C.R. Liver and spleen bacterial loads in Lcn2^fl/fl^Rorc^cre^ mice were also significantly increased (Fig. 4h, i). In terms of cell phenotypes, C.R infection did not significantly alter the proportions and absolute numbers of CD4^+^RORγt^+^ cells in LPMCs from Lcn2^fl/fl^Rorc^cre^ mice compared with those in Lcn2^fl/fl^ mice (Supplementary Fig. 8a, Fig. 4j). However, significant reductions in the proportions and numbers of ILC3s from mLNs (Supplementary Fig. 8b, c) and LPMCs (Supplementary Fig. 8d, Fig. 4k) were observed in Lcn2^fl/fl^Rorc^cre^ mice, indicating that LCN2 specifically regulates ILC3s independently of T cells, similar to GPX4. Consistently, Lcn2^fl/fl^Rorc^cre^ mice exhibited notable decreases in the proportions and numbers of NKp46^+^ILC3 and DN ILC3 subsets in LPMCs, whereas those of CCR6^+^ILC3 cells remained unchanged (Supplementary Fig. 8d, Fig. 4l-n). Similar to those in Gpx4^fl/fl^Rorc^cre^ mice, the proportions and absolute numbers of IL-22^+^ILC3s and IL-17A^+^ILC3s in LPMCs of Lcn2^fl/fl^Rorc^cre^ mice were significantly diminished (Supplementary Fig. 8e, Fig. 4o, p), whereas those of intestinal neutrophils were markedly increased (Fig. 4q). Concurrently, secretion of the antimicrobial peptides RegIIIβ and RegIIIγ by IECs was substantially reduced (Fig. 4r), indicating exacerbated intestinal inflammation.

To elucidate the precise regulatory mechanism underlying ferroptosis in ILC3s during colitis, we isolated ILC3s from LPMCs of Lcn2^fl/fl^ and Lcn2^fl/fl^Rorc^cre^ mice after C.R infection, followed by a transcriptomic analysis. Our results revealed a significant upregulation of ferroptosis markers including prostaglandin endoperoxide synthase 2 (*Ptgs2*) and acyl CoA chain member 4 synthase (*Acsl4*) in ILC3s derived from the intestines of Lcn2^fl/fl^Rorc^cre^ mice compared with that in Lcn2^fl/fl^ mice. Furthermore, the expression of critical negative regulators, such as *Atf4* and its downstream targets *xCT* (*Slc7a11*), *Slc3a2*, and *Gpx4*, involved in ferroptosis, was significantly downregulated (Fig. 4s). RT-qPCR data confirmed these changes (Fig. 4t), verifying the inhibitory effect of LCN2 on ferroptosis in ILC3s. Consistently, the ferroptosis characteristics of intestinal ILC3s in Lcn2^fl/fl^Rorc^cre^ mice were markedly enhanced, including a substantial reduction in cell viability (Fig. 4u), an elevation in intracellular lipid ROS accumulation (Fig. 4v), and an increase in Fe^2+^ content (Fig. 4w). Additionally, protein expression of the transcription factor ATF4 (Fig. 4x) and its downstream target xCT (Fig. 4y), as well as GPX4 (Fig. 4z), were significantly reduced in the ILC3s of Lcn2^fl/fl^Rorc^cre^ mice compared with those in Lcn2^fl/fl^ mice. Collectively, these findings suggest that LCN2 governed the functionality of ILC3s and their susceptibility to ferroptosis in colitis. This regulatory mechanism potentially involves ATF4 and its downstream target xCT, along with GPX4.

### LCN2 mediates ferroptosis resistance in ILC3s via the ATF4-xCT/GPX4 axis

**Fig. 5:**
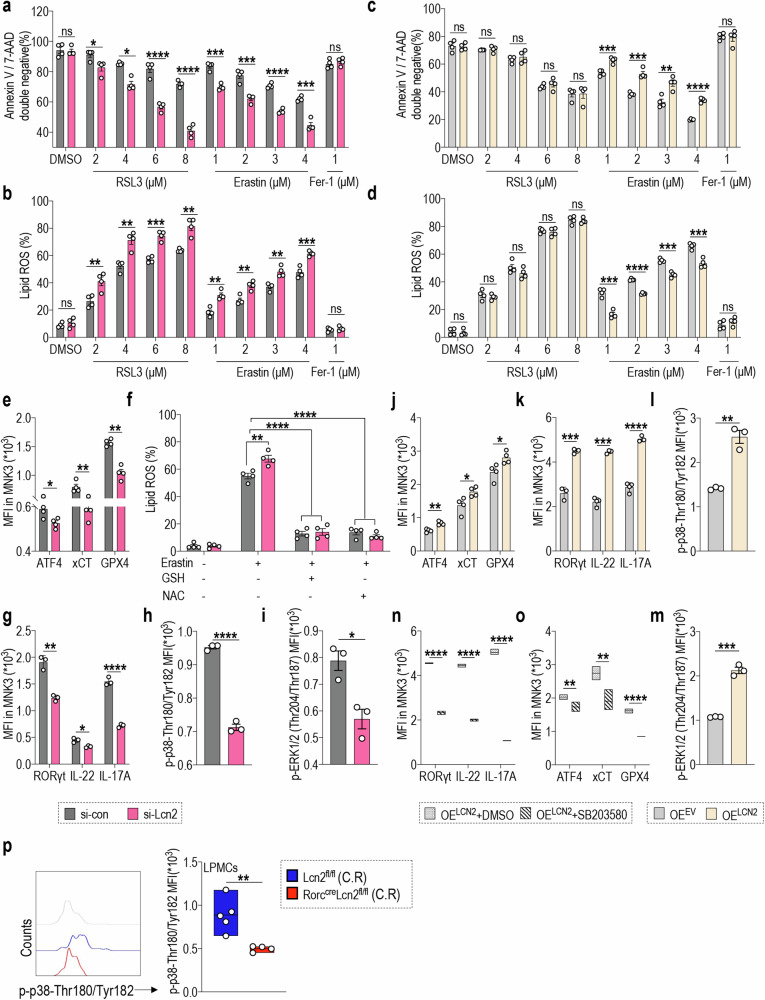
LCN2 mediates ferroptosis resistance in ILC3s via the ATF4-xCT/GPX4 axis. MNK3 cells were transfected with si-con / si-Lcn2, or plasmids encoding LCN2 (OE^LCN2^) / empty vector (OE^EV^) and treated with RSL3 (2-8 μM), erastin (1–4 μM) or vehicle (DMSO) for 16 h. Statistical results of **a**, **c** the percentage of annexin V and 7-AAD double-negative population and **b**, **d** lipid ROS production in the indicated MNK3 cells. (*n* = 4) **e** ATF4, xCT, and GPX4 expression in MNK3 cells following transfection with si-con or si-Lcn2 and treatment with erastin for 16 h. (*n* = 4) **f** After transfecting MNK3 cells with si-con or si-Lcn2, the MNK3 cells were subjected to erastin (4 μM) treatment for 12 h in the presence or absence of GSH (0.5 mM) or NAC (0.5 mM), and subsequently, lipid ROS production was assayed. (*n* = 4) **g** RORγt, IL-22, and IL-17A expression in MNK3 cells following transfection with si-con or si-Lcn2 and treatment with erastin for 16 h. (*n* = 3) Statistical results of MFI for **h** p-p38-Thr180/Tyr182 and **i** p-ERK1/2 (Thr204/Thr187) expression in the indicated MNK3 cells following treatment with erastin. (*n* = 3) **j** ATF4, xCT, and GPX4 expression in MNK3 cells following transfection with plasmids OE^LCN2^ or OE^EV^ and treatment with erastin for 16 h. (*n* = 4) **k** RORγt, IL-22, and IL-17A expression was assessed in indicated MNK3 cells treated with erastin for 16 h. (*n* = 3) Statistical results of MFI for **l** p-p38-Thr180/Tyr182 and **m** p-ERK1/2 (Thr204/Thr187) expression in indicated MNK3 cells after treatment with erastin. (*n* = 3) **n**, **o** After transfecting MNK3 cells with plasmids OE^LCN2^ or OE^EV^, the cells were exposed to erastin for 16 h in the presence or absence of the p38 inhibitor SB203580. **n** RORγt, IL-22, and IL-17A expression in indicated MNK3 cells. (*n* = 3) **o** ATF4, xCT, and GPX4 expression in indicated MNK3 cells. (*n* = 3) **p** Statistical results of MFI for p-p38-Thr180/Tyr182 expression in ILC3s from LPMCs of Lcn2^fl/fl^ and Rorc^cre^Lcn2^fl/fl^ mice post C.R infection. (n = 4) Data are presented as the mean ± SEM or median, and statistical significance was determined using two-sided unpaired *t*-test (**a**–**p**). *P < 0.05; **P < 0.01; ***P < 0.001.

To determine the molecular network regulated by LCN2 in ILC3s, we used the MNK3 cell line. We employed specific small interfering RNA (siRNA) targeting LCN2 (si-Lcn2) to effectively suppress LCN2 expression in MNK3 cells, resulting in a significant reduction in both LNC2 protein and mRNA levels compared with those in the control group (si-con) (Supplementary Fig. 9a, b); the secretion of IL-22 and IL-17A was also significantly reduced (Supplementary Fig. 9c, d), indicating that LCN2 exerted a positive regulatory effect on MNK3 cell functions. To investigate the effect of LCN2 on ferroptosis, MNK3 cells were treated with two distinct ferroptosis inducers, RSL3 [42] and erastin [22], and cultured in vitro. The suppression of LCN2 expression in MNK3 cells significantly enhanced the classical manifestations of ferroptosis induced by RSL3 or erastin, characterized by a substantial reduction in cell viability and augmented intracellular lipid ROS accumulation (Fig. 5a, b). To validate the specific enhancement of ferroptosis with LCN2 inhibition, we employed Fer-1, a selective inhibitor of ferroptosis. Fer-1 treatment completely reversed the ferroptosis characteristics induced by RSL3 and erastin in both si-con and si-Lcn2 groups. Thus, inhibiting LCN2 expression in MNK3 cells significantly augmented ferroptosis induction.

Next, we assessed the effect of LCN2 overexpression on MNK3 cells. In MNK3 cells transfected with a plasmid encoding LCN2 (OE^LCN2^), both protein and mRNA levels were elevated (Supplementary Fig. 9e, f), leading to the significantly enhanced secretion of IL-22 and IL-17A (Supplementary Fig. 9g, h). However, LCN2 overexpression in MNK3 cells only diminished erastin-induced ferroptosis without reversing the RSL3-induced decrease in cell viability, accumulation of lipid ROS, and subsequent ferroptosis (Fig. 5c, d). Considering the reported binding of RSL3 to GPX4 and their inhibition of GPX4 activity to trigger ferroptosis, our data suggest that LCN2 exerts its role upstream of GPX4 [42]. Notably, LCN2 inhibition in MNK3 cells also significantly downregulated ATF4, xCT, and GPX4 expression after erastin treatment (Fig. 5e).

Additionally, upon investigating the potential mechanisms, the pre-treatment of cells with exogenous GSH or N-acetylcysteine (NAC) completely reversed erastin-induced lipid ROS accumulation in both the si-con and si-Lcn2 groups of MNK3 cells (Fig. 5f). The inhibition of LCN2 expression in MNK3 cells significantly reduced RORγt, IL-22, and IL-17A expression after erastin treatment (Fig. 5g). Meanwhile, a notable decrease was observed in the phosphorylation levels of p38 (Fig. 5h) and ERK1/2 (Fig. 5i), indicating impaired MNK3 cell functionality. Conversely, following erastin treatment, the overexpression of LCN2 in MNK3 cells significantly reinstated the expression of ATF4, xCT, and GPX4 (Fig. 5j) while inducing an increase in RORγt, IL-22, and IL-17A expression (Fig. 5k). Concomitantly, there was a significant elevation in phosphorylation levels of p38 (Fig. 5l) and ERK1/2 (Fig. 5m), indicating restoration of MNK3 cell function.

To elucidate the specific signaling pathway involved in LCN2, we used the p38 inhibitor SB203580 [58] and ERK1/2 inhibitor U0126. As anticipated, SB203580 and U0126 significantly attenuated the expression of RORγt, IL-22, and IL-17A in the control (OE^EV^) and OE^LCN2^ groups (Fig. 5n, Supplementary Fig. 10a–d). Notably, LCN2 overexpression in MNK3 cells combined with SB203580 treatment showed a significant reduction in ATF4, xCT, and GPX4 expression (Fig. 5o). Conversely, U0126 did not impact ATF4, xCT, or GPX4 expression (Supplementary Fig. 10e). Importantly, no alterations were detected in ATF4, xCT, and GPX4 expression when either SB203580 or U0126 was administered to the OE^EV^ group of MNK3 cells (Supplementary Fig. 10f–h). Consistently, the p38 phosphorylation levels in the ILC3s of Lcn2^fl/fl^Rorc^cre^ mice were significantly decreased after C.R infection compared with those in the Lcn2^fl/fl^ mice (Fig. 5p).

Together, we identified LCN2 as a negative regulator of ferroptosis, thereby providing novel insights into the mechanisms associated with ferroptosis in ILC3s. Specifically, our findings indicate that LCN2 mediated the regulatory effect of ILC3s on colitis through the ATF4-xCT/GPX4 axis. Notably, p38 signaling can specifically function downstream of LCN2 to modulate ferroptosis functionality and susceptibility in ILC3s.

### p38 signaling downstream of LCN2 is specifically involved in regulating NKp46^+^ILC3 functions and susceptibility to ferroptosis during colitis

**Fig. 6:**
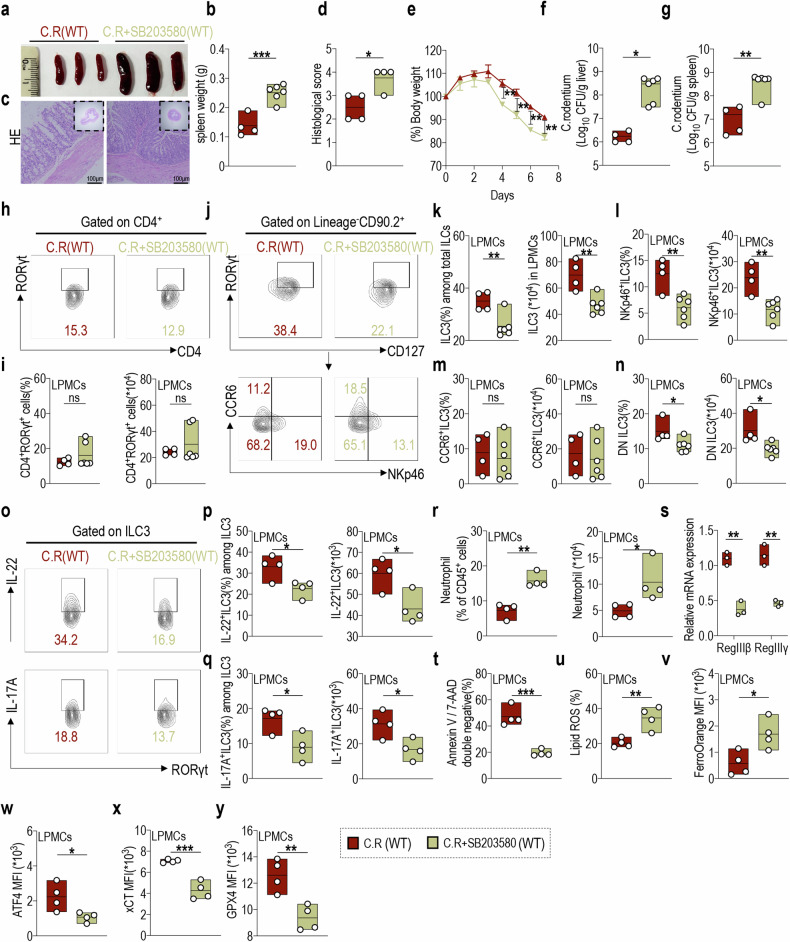
p38 signaling downstream of LCN2 is specifically involved in regulating NKp46^+^ILC3 functions and susceptibility to ferroptosis during colitis. The WT mice were orally administered 1 × 10^8^ CFU of C.R and treated with or without the p38 inhibitor SB203580. Mouse tissues were collected on day 8 post infection (1 mg/kg/day, i.p.). **a** Representative spleen images and **b** statistical results of spleen weights at day 8 post infection. (*n* = 4) **c** Representative H&E-stained colon sections from indicated mice (scale bars: 100 μm). **d** The statistical results of histological scores are shown. (*n* = 4) **e** The body weight change rates of indicated mice. (*n* = 4) Log_10_ CFU of C.R in **f** liver and **g** spleen tissues of indicated mice on day 8 post infection. (*n* = 4) **h** The representative flow cytometry plots of CD4^+^RORγt^+^ cells in LPMCs from the indicated mice. **i** Statistical results of proportion (left) and numbers (right) of CD4^+^RORγt^+^ cells are shown. (*n* = 4) **j** The representative flow cytometry plots and statistical results of the proportion (left) and number (right) of **k** ILC3s, including **l** NKp46^+^ILC3, **m** CCR6^+^ILC3, and **n** DN cell subsets in LPMCs of indicated mice. (*n* = 4) **o** Representative flow cytometry plots of IL-22- (upper) and IL-17A-positive (lower) ILC3s in LPMCs. Statistical results of proportion (left) and absolute numbers (right) of **p** IL-22-, and **q** IL-17A-expressing ILC3 are shown. (*n* = 4) **r** Statistical results of the proportion (left) and numbers (right) of neutrophils in LPMCs from the indicated mice. (*n* = 4) **s** Relative RegIIIβ and RegIIIγ mRNA expression in the colon tissue of indicated mice. (*n* = 3) Statistical results of **t** the percentage of annexin V and 7-AAD double-negative population and **u** lipid ROS production in LPMCs-derived ILC3s from the indicated mice. (*n* = 4) **v** Fe^2+^ level in ILC3s from LPMCs was analyzed using FerroOrange, and the statistical results are shown. (*n* = 4) The expression levels of **w** ATF4, **x** xCT, and **y** GPX4 in ILC3s from LPMCs of indicated mice. (*n* = 4) Data are presented as the mean ± SEM or median, and statistical significance was determined using two-sided unpaired *t-*test (**b**, **d**–**g**, **i**, **k**–**n**, **p**–**y**). *P < 0.05; **P < 0.01; ***P < 0.001.

To elucidate the specificity of p38 signaling in terms of the molecular mechanism underlying LCN2 regulation, we used the C.R infection model with WT mice and administered daily p38 inhibitor (SB203580) treatments, followed by subsequent analysis after 8 days. SB203580-treated C.R infected mice exhibited exacerbated intestinal inflammation, consistent with observations in C.R infected Gpx4^fl/fl^Rorc^cre^ mice and Lcn2^fl/fl^Rorc^cre^ mice. Specifically, the spleen weight of mice infected with C.R and treated with SB203580 exhibited a significant increase (Fig. 6a, b). The colonic pathological score was also elevated (Fig. 6c, d), accompanied by a substantial decrease in body weight starting from day 4 post C.R infection (Fig. 6e). The bacterial load in the livers and spleens of C.R-infected mice treated with SB203580 also increased (Fig. 6f, g).

Regarding the cell phenotype, no significant alterations in the proportions and absolute numbers of CD4^+^RORγt^+^ cells in LPMCs from mice infected with C.R and treated with SB203580 were observed (Fig. 6h, i). However, a decrease in the proportion and number of intestinal LPMC-derived ILC3s was observed (Fig. 6j, k). These findings were consistent with observations from Lcn2^fl/fl^Rorc^cre^ mice. Additionally, treatment with SB203580 significantly reduced the proportions and number of NKp46^+^ILC3 and DN ILC3 subsets in LPMCs from C.R-infected mice, whereas no significant changes were observed in CCR6^+^ILC3s (Fig. 6l–n). Meanwhile, after SB203580 treatment, a substantial reduction was noted in both the proportions and absolute numbers of IL-22^+^ILC3s and IL-17A^+^ILC3s among LPMCs from C.R-infected mice (Fig. 6o–q). In contrast, intestinal neutrophil numbers were significantly increased (Fig. 6r). Additionally, secretion of the antimicrobial peptides RegIIIβ and RegIIIγ was markedly reduced (Fig. 6s), indicating exacerbated intestinal inflammation.

Importantly, as observed in the intestinal ILC3s of Lcn2^fl/fl^Rorc^cre^ mice, the ferroptosis characteristics of C.R-infected mouse intestinal ILC3s were significantly enhanced after SB203580 treatment, as evidenced by a notable decrease in cell viability (Fig. 6t), an elevation in intracellular lipid ROS accumulation (Fig. 6u), and an augmentation in Fe^2+^ content (Fig. 6v). Furthermore, SB203580 treatment markedly attenuated the expression of transcription factor ATF4 (Fig. 6w) and its downstream targets xCT (Fig. 6x) and GPX4 (Fig. 6y) in ILC3s of LPMCs. Overall, these data further validated the role of the p38 signaling pathway as a specific regulator downstream of LCN2 in modulating the ATF4-xCT/GPX4 axis, thereby positively influencing ILC3 function and negatively regulating ferroptosis characteristics in ILC3s, thus highlighting its protective effects against mouse colitis.

### Restoring GPX4 expression through Fer-1 treatment effectively alleviates colitis signs in Lcn2^fl/fl^Rorc^cre^ mice

Herein, we substantiated the specific regulatory effect of LCN2 on mouse colitis through the inhibition of ILC3 ferroptosis and promotion of ILC3 functionality. However, we also sought to determine whether disrupting ferroptosis could directly protect mice from the development of colitis. Therefore, we treated C.R infection model Lcn2^fl/fl^Rorc^cre^ mice with the ferroptosis inhibitor Fer-1 [57]. With this intervention, we aimed to demonstrate the contribution of ferroptosis to severe intestinal inflammation in Lcn2^fl/fl^Rorc^cre^ mice infected by C.R.

**Fig. 7:**
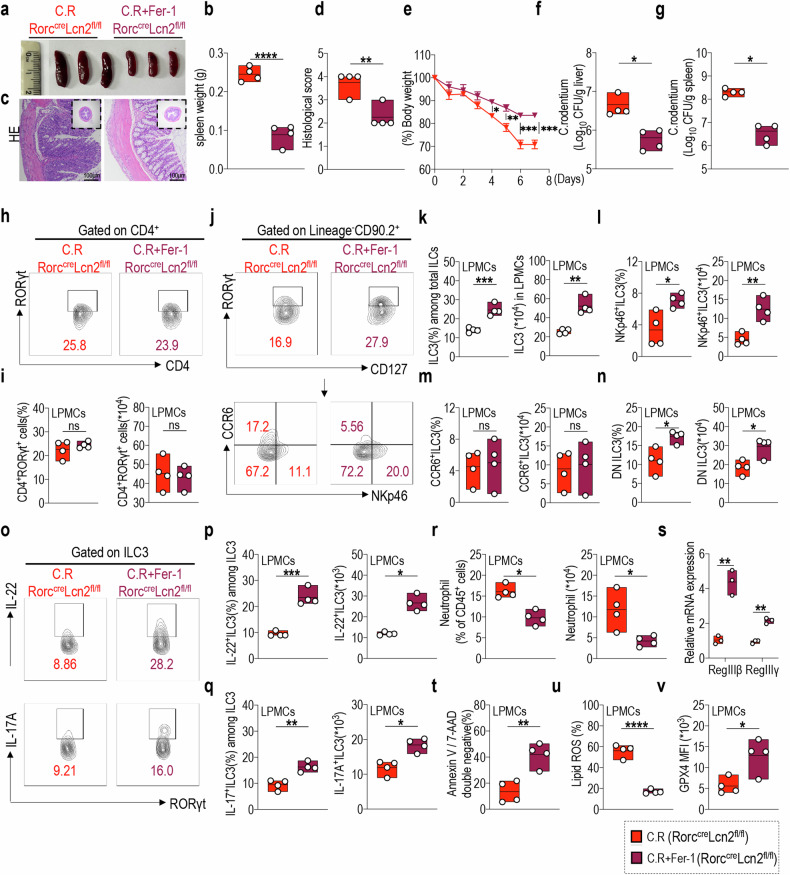
Restoring GPX4 expression through Fer-1 treatment effectively alleviates colitis signs in Lcn2^fl/fl^Rorc^cre^ mice. Rorc^cre^Lcn2^fl/fl^ mice infected with C.R were treated with or without Fer-1 (10 mg/kg/day, i.p.), and tissue samples were collected on day 8. **a** Representative spleen images and **b** statistical results of spleen weight of the indicated mice. (n = 4) **c** Representative H&E-stained colon sections from indicated mice (scale bars: 100 μm). **d** The statistical results of histological scores are shown. (*n* = 4) **e** Body weight changes of the indicated mice. (*n* = 4) Log10 CFU of C.R in **f** liver and **g** spleen tissues from indicated mice on day 8 post infection. (*n* = 4) **h** Representative flow cytometry plots and **i** statistical results of the comparison of CD4^+^RORγt^+^ cell numbers in LPMCs of indicated groups of mice. (*n* = 4) **j** Representative flow cytometry plots and statistical results of the comparison of **k** ILC3s, including **l** NKp46^+^ILC3, **m** CCR6^+^ILC3, and **n** DN cell populations in LPMCs of indicated mice. (n = 4) **o** Representative flow cytometry plots and statistical results of the comparison of **p** IL-22-, and **q** IL-17A-positive ILC3s from LPMCs of the indicated mice. (*n* = 4) **r** Statistical results of the proportion (left) and numbers (right) of neutrophils in LPMCs from the indicated mice. (*n* = 4) **s** Relative RegIIIβ and RegIIIγ mRNA expression in the colon tissue of the indicated mice. (*n* = 3) Statistical results of **t** the percentage of annexin V and 7-AAD double-negative population and **u** lipid ROS production in LPMCs-derived ILC3s from the indicated mice. (*n* = 4) **v** Statistical results of GPX4 expression in ILC3s from LPMCs of the indicated mice. (*n* = 4) Data are presented as the mean ± SEM or median, and statistical significance was determined using two-sided unpaired *t*-test (**b**, **d**–**g**, **i**, **k–n**, **p**–**v**). *P < 0.05; **P < 0.01; ***P < 0.001.

Treatment with Fer-1 effectively ameliorated intestinal inflammation in Lcn2^fl/fl^Rorc^cre^ mice infected with C.R. Notably, the administration of Fer-1 significantly reduced spleen weight (Fig. 7a, b), accompanied by a decrease in colonic pathological score (Fig. 7c, d) in Lcn2^fl/fl^Rorc^cre^ mice following C.R infection. Weight recovery was also observed from day 4 post C.R infection in Fer-1 treated Lcn2^fl/fl^Rorc^cre^ mice (Fig. 7e). Fer-1 treatment significantly reduced bacterial load in the livers and spleens of Lcn2^fl/fl^Rorc^cre^ mice post-C.R infection (Fig. 7f, g).

Regarding changes in cell phenotype, no significant alterations in the proportion and absolute number of CD4^+^RORγt^+^ cells in LPMCs from C.R-infected Lcn2^fl/fl^Rorc^cre^ mice treated with Fer-1 (Fig. 7h. i); however, the number of resident ILC3s in LPMCs was increased (Fig. 7j, k), especially for NKp46^+^ILC3 and DN ILC3 subsets, whereas no significant change was observed in CCR6^+^ILC3s (Fig. 7l-n). Additionally, Fer-1 treatment restored the proportions and absolute numbers of IL-22^+^ILC3s and IL-17A^+^ILC3s in the LPMCs from C.R-infected Lcn2^fl/fl^Rorc^cre^ mice (Fig. 7o-q), leading to a reduction in the number of intestinal neutrophils (Fig. 7r). Simultaneously, the secretion of the antimicrobial peptides RegIIIβ and RegIIIγ by IECs was significantly increased, indicating the effective alleviation of intestinal inflammation (Fig. 7s).

Moreover, after Fer-1 treatment, a pronounced reduction in the ferroptosis characteristics of LPMC-derived ILC3s from Lcn2^fl/fl^Rorc^cre^ mice infected with C.R was observed, including significantly enhanced cell viability (Fig. 7t) and decreased intracellular lipid ROS accumulation (Fig. 7u). Furthermore, targeted therapy with Fer-1 restored the expression of GPX4 (Fig. 7v) in ILC3 of LPMCs from C.R-infected Lcn2^fl/fl^Rorc^cre^ mice; however, no substantial alterations were observed in its upstream signals, including LCN2 protein expression, p38 phosphorylation level, ATF4 expression, and downstream xCT expression (Supplementary Fig. 11a–d). The experimental results with Fer-1 treatment demonstrated that the restoration of GPX4 expression and disruption of ferroptosis effectively ameliorated colitis symptoms in Lcn2^fl/fl^Rorc^cre^ mice.

## Discussion

**Fig. 8:**
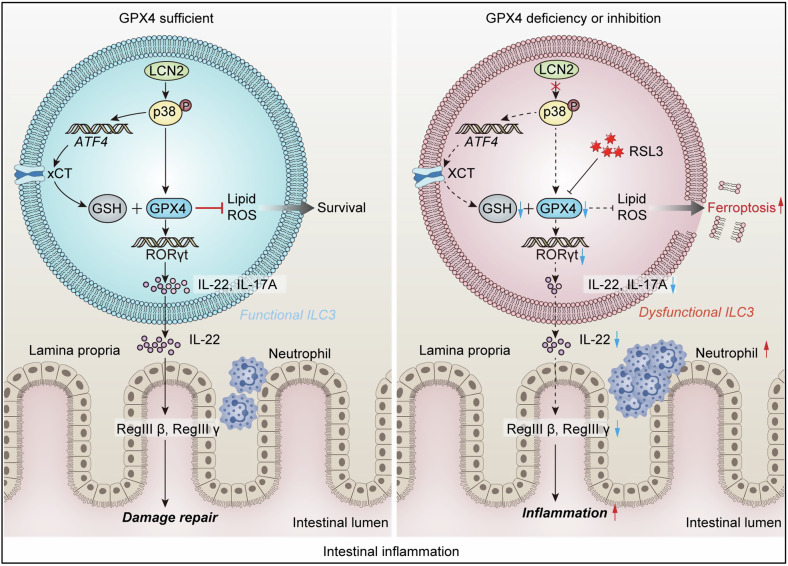
Working model of the regulatory role of LCN2-ATF4-xCT/GPX4 axis in modulating intestinal ILC3 cell function and susceptibility to ferroptosis.

ILC3s play a crucial role in regulating intestinal homeostasis. Intestinal homeostasis imbalances have been closely associated with UC pathogenesis, and emerging evidence suggests a link between ferroptosis and the etiology of UC [22, 34, 35]. However, the precise coordination of ferroptosis-related molecular mechanisms with the immune response of ILC3s in the intestinal mucosal barrier remains largely unknown. Herein, we discovered that intestinal inflammation induces upregulation of LCN2 expression in ILC3s, particularly within the NKp46^+^ILC3 subpopulation, which activates the p-p38-ATF4-xCT axis and enhances GPX4 expression to inhibit ferroptosis in ILC3s, thereby promoting ILC3 activation. Accordingly, the levels of IL-22 and IL-17A in activated ILC3s were elevated, facilitating mucosal barrier repair and alleviating intestinal inflammation. Conversely, LCN2 or GPX4 deficiency or GPX4 expression inhibition by the ferroptosis inducer RSL3 rendered mouse intestinal ILC3s susceptible to ferroptosis, impairing the secretion of IL-22 and IL-17A, which are required for mucosal barrier repair functions. Additionally, the release of the antimicrobial peptides RegIII β and RegIII γ by IECs was reduced, and neutrophil recruitment was increased, thereby exacerbating mouse colitis (Fig. 8).

Research on ferroptosis in patients with UC is in its nascent stage, and numerous areas worthy of exploration remain. Previous extensive studies have predominantly established the significance of ferroptosis in the pathophysiology of UC based on intestinal disease phenotypes. Ferroptosis primarily occurs in IECs, resulting in IEC death and epithelial erosion [34, 59], thereby mediating abnormal inflammation and disease progression in UC [60]. Chen et al. demonstrated that inhibiting ferroptosis improved intestinal symptoms in UC mice [61]. However, knowledge regarding the specific effects of ferroptosis on immune cells within the context of UC is lacking. Ferroptosis is involved in modulating macrophage proportions and thus regulating UC in a colitis model [62]. This study presents compelling evidence for the regulation of innate immune cells through ferroptosis in UC. Here, we not only validated the beneficial impact of ferroptosis inhibition on UC regulation but also discovered, for the first time, that ILC3s, as pivotal innate immune cells, have a critical role in modulating UC disease progression via functional alterations and susceptibility to ferroptosis.

We systematically investigated the regulatory role of ferroptosis in intestinal ILC3s in UC progression and established a correlation between ferroptosis in ILC3s, particularly NKp46^+^ILC3s, and their secretion of IL-22 and IL-17A for mucosal barrier repair and regulation of colitis through the application of ferroptosis inducers or inhibitors in mice. NKp46^+^ILC3s are a crucial source of tissue-protective IL-22 and are essential in maintaining intestinal homeostasis [63, 64]. The absence of these cells is associated with increased severity of acute colitis [64], which was further corroborated by our research. We also conducted an in-depth investigation into the specific molecular network underlying the involvement of ILC3 cell ferroptosis in regulating their functionalities within the context of UC. Ferroptosis disrupts the intestinal barrier and contributes to the pathogenesis of DSS-induced colitis [65]. GPX4, a critical suppressor of ferroptosis, is a crucial target for investigating the involvement of ferroptosis in colitis pathogenesis. However, current research on the relationship between GPX4 and UC has mainly focused on pathological phenotypic changes. Restoring GPX4 expression can alleviate ferroptosis and mitigate pathological changes in mouse colitis [61]. Nevertheless, research regarding the correlation between the mechanisms of ferroptosis and immune cell functional alterations in UC is limited.

Here, we employed GPX4 conditional-knockout mice with specific ablation of GPX4 expression in the crucial transcription factor RORγt within the intestinal tissue to investigate the precise regulatory impact of GPX4 on intestinal inflammation for the first time. Our findings indicated that GPX4 mediated ILC3s, particularly the NKp46^+^ILC3 subpopulation, specifically regulating colitis, accompanied by alterations in ferroptosis characteristics of ILC3s and their secretion capacity for IL-22 and IL-17A. To fully elucidate the role of GPX4 in this process, recovery experiments using inhibitors targeting ferroptosis pathways should be conducted in the future. Notably, our results suggest that T cells did not significantly contribute to the overall regulatory process; however, further comprehensive investigation utilizing T cell knockout mouse models is required to substantiate this assertion.

Our findings suggest that LCN2 specifically regulated ILC3s, particularly the NKp46^+^ILC3 subgroup, to secrete IL-22 and IL-17A by targeting GPX4, thereby exerting a protective effect on colitis. This result holds promise for ameliorating intestinal inflammation and promoting mucosal healing in IBD. Conversely, the plasticity of ILC3s induced by mucosal bacterial infection can facilitate the conversion of ILC3s to ILC1s, thereby leading to IFNγ-dependent intestinal pathology [66]. However, we did not fully determine whether LCN2 mediated the plasticity of ILC3s. Further elucidation of the precise impact of the molecular regulation mechanism of LCN2 in ILC3s on the plasticity of ILC subpopulations will facilitate the optimization of targeted therapeutic strategies for ILC responses in intestinal diseases, which constitutes our forthcoming research focus. Moreover, IL-22, derived from ILC3s, is crucial in regulating LCN2 production in human IECs, thereby facilitating host defense against Enterobacteriaceae infections [67]. Our findings provide novel insights into the extent to which IL-22 governs antibacterial responses and the involvement of ILC3s in modulating LCN-2 expression in human IECs. Our research aimed to investigate the impact of endogenous alterations in LCN2 expression on the regulatory function of ILC3s under normal and colitis-related conditions. However, whether secreted LCN2 influences extracellular interactions with ILC3s remains unclear. A comprehensive investigation into the crosstalk between ILC3-secreted LCN2 and IECs and the associated specific mechanisms would be intriguing.

Previous studies have reported close association between gut microbiota dysbiosis and UC pathogenesis [68, 69] and established a correlation between gut microbiota metabolites and ferroptosis [70, 71]. Our findings suggest that the LCN2-p38-ATF4-xCT/GPX4 pathway mediated the susceptibility of ILC3s to ferroptosis; however, we are yet to elucidate the specific alterations in the gut microbiota involved. Therefore, our forthcoming research will focus on studying the relationship between changes in short-chain fatty acids and gut microbiota through high-throughput 16S rRNA sequencing and targeted metabolomics to establish a potential link with the regulatory mechanism of ferroptosis molecules in the progression of UC.

In conclusion, our study validated GPX4 as a novel molecular regulator of ILC3 function. Activation of the LCN2-p38-ATF4-xCT/GPX4 axis attenuated the susceptibility of ILC3s to ferroptosis and enhanced their capacity for IL-22 and IL-17A secretion. This specific mechanism effectively contributed to maintaining intestinal homeostasis and prevented colitis development in mice. Our research established a fundamental basis for investigating the correlation between the regulatory mechanism of ferroptosis in immune cells and disease regulation, thereby bridging the existing gap in understanding the relationship between ILC3s and ferroptosis in UC. This study provides valuable insights into strategies aimed at modulating intestinal homeostasis.

## Materials and methods

### Antibodies and reagents

Details of the antibodies and reagents used in this study are listed in the Supporting Information Tables S1 and S2, respectively.

### Human samples

Colonic mucosa tissues were provided by the Department of Gastroenterology of the Sixth Affiliated Hospital of Sun Yat-sen University (Guangzhou, China). All samples were obtained from participants undergoing colonoscopy, and patients who had received any type of treatment in the past 3 months were excluded. UC was diagnosed based on the gold standard of endoscopic examination and biopsy pathology evaluation. Participants without a diagnosis of UC or other intestinal diseases were considered as HCs. The corresponding research plan and ethical approval were provided by the Clinical Trial Ethics Committee of the Sixth Affiliated Hospital of Sun Yat-sen University (Approval ID: E2022243). Written informed consent was obtained from all participants or their legal guardians at the time of admission.

### Mice

C57BL/6 J mice (male and female) were purchased from the Laboratory Animal Center of Southern Medical University (Guangzhou, China). Lcn2^fl/fl^ mice were generated by Cyagen Bioscience (Guangzhou, China). Gpx4^fl/fl^ mice were generated by Shanghai Model Organisms Center, Inc (Shanghai, China). Rorc^cre^ mice were kind gifts from professor C. Dong from Tsinghua University. All transgenic mice were bred and maintained in the animal facility of the Laboratory Animal Center of Southern Medical University under specific pathogen-free conditions. Age- and sex-matched littermates between 8 and 16 weeks of age were used in all experiments. Both male and female mice were used in experiments and were assigned randomly to experimental groups. All experimental protocols were approved by the Institutional Animal Care and Use Committee of Southern Medical University Experimental Animal Ethics Committee (Approval number: L2021072).

### Preparation of mouse tissue samples

Mice were euthanized, followed by cervical dislocation. The mLNs and intestines were removed from the sacrificed mice and washed in ice-cold phosphate-buffered saline (PBS). The intestines were cut longitudinally and thoroughly cleaned in ice-cold PBS, and mechanically cut into 1 cm pieces using scissors. Next, intestinal pieces were transferred to HBSS buffer containing 10 mM EDTA (Promega, Madison, WI, USA) and 1 mM dithiothreitol (Fermentas, Waltham, MA, USA) for a 30-min incubation at 37 °C on a horizontal shaker to remove epithelial cells and mucus. After the tissues were vortexed and washed twice with PBS, the epithelial fractions were discarded. The remaining intestinal tissues were cut into smaller 1 mm pieces and digested in Roswell Park Memorial Institute (RPMI) 1640 containing 5% fetal bovine serum, 1 mg/mL collagenase I (Sigma-Aldrich, St. Louis, MO, USA), 100 μg/mL DNase I (Sigma-Aldrich), 1 mg/mL dispase (Roche, Basel, Switzerland), and 10 mM HEPES for a 45-min incubation at 37 °C on a horizontal shaker. Next, the digested tissues were passed through a 70 μm cell strainer, and resuspended in 40% Percoll (GE Healthcare, Chicago, IL, USA). LPMCs were enriched with 40% / 80% Percoll gradient. After centrifugation at 37 °C, 400 × *g* for 25 min, the white membrane was aspirated and washed twice with PBS to obtain the purified LPMCs for flow cytometry analysis or sorting. The mLNs were mechanically ground and passed through a 70 μm cell filter to harvest single-cell suspension for subsequent experiments.

### Flow cytometry

For flow cytometry analysis, single-cell suspensions were surface-stained with fluorophore-conjugated antibodies for 40 min on ice. For intracellular staining, isolated cells were surface-stained, fixed, and permeabilized within a Foxp3/Transcription Factor Staining Buffer (TONBO Biosciences). Subsequently, the cells were labeled with intracellular antibodies for 30 min at room temperature. For measuring intracellular cytokines (IL-17A, and IL-22), the cells were stimulated with 50 ng/mL phorbol-12-myr-sitastate 13 acetate (Alomone), 1 μg/mL ionomycin (Alomone), and 1 μg/mL Brefeldin A (Alomone) in completed RPMI1640 media at 37 °C, 5% CO_2_ for 5 h before staining. The following antibodies were used for flow cytometry analysis of ILC3: CD45^+^CD4^-^ Lin (CD3, B220, CD11b, Ly6G, TER-119, CD11c, CD5, CD8a, TCRβ, TCRγδ)^-^ CD90.2^+^ CD127^+^ RORγt^+^. The following antibodies were used to label the ILC3s for sorting of flow cytometry: Lin (CD3, B220, CD11b, Ly6G, TER-119, CD11c, CD5, CD8a, TCRβ, TCRγδ)^-^ CD45^low^CD90.2^hi^CD127^+^.

For 7-AAD and annexin V staining, cells were harvested and resuspended in 100 μL of Annexin V Binding Buffer with APC Annexin V and 7-AAD Viability Staining Solution (BioLegend, 640930). The cells were then incubated at room temperature in the dark for 15 min. A total of 200 μL of Annexin V Binding Buffer was added to each tube before flow cytometry analysis.

FACS data were acquired using LSR Fortessa flow cytometer (BD Biosciences) and analyzed using the FlowJo V10.0.8 software. The flow cytometry was performed at the Department of Immunology and Department of Developmental Biology at the School of Basic Medical Sciences, Southern Medical University.

### *C. rodentium* infection model

*C. rodentium* (C.R) (DBS100, ATCC 51459) was cultured in Luria–Bertani broth overnight at 37 °C with horizontal shaking at 200 rpm. Subsequently, the bacteria were centrifuged after 16 h, the supernatant was discarded, and the remaining bacterial precipitate was resuspended in sterile PBS. Mice were inoculated with 1 × 10^8^ colony-forming units (CFU) of C.R in 200 μL of PBS by oral gavage after an 8 h fast. Quantification of C.R was performed by serial diluting and culturing overnight on MacConkey agar plates at 37 °C. The mice were weighed daily and sacrificed for analysis on day 8 post infection. The intestine was removed, and its length was measured. To assess bacterial load, spleens and livers of infected mice were collected, weighed, and homogenized. Serial dilutions of the homogenate were plated onto MacConkey agar plates, and the number of CFUs was measured after overnight incubation at 37 °C.

### Enzyme-linked immunosorbent assays (ELISA)

ELISA kits (4 A BIOTECH, Beijing, China) were used to measure cytokine levels in the cell culture supernatants. All procedures were performed according to the manufacturer’s instructions and all measurements were conducted using a Thermo Fisher Scientific Multiskan FC system.

### RNA extraction and RT-qPCR analysis

Total RNA was extracted using TRIzol (Catalog No. 15596; Invitrogen, Carlsbad, CA, USA), and reverse transcription was performed using a StarScript II First-strand cDNA Synthesis Kit (catalog no. A212-05; GenStar, Beijing, China). RT-qPCR analysis was performed using the RealStar Green Power Mixture kit (Catalog No. A314-10; GenStar) on the QuantStudio 6 Flex system (Thermo Fisher Scientific), according to the manufacturer’s instructions. The relative expression data were normalized to the expression of *β-actin* (reference gene) in all groups, and the lowest expression in the control group was artificially set to 1. All experiments were repeated at least twice. The primer sequences used in this study are listed in Supporting Information Table S3.

### ILC3 isolation

ILC3s were isolated from the intestine of mice using flow cytometry (ILC3 = Lin^-^ CD45^low^CD90.2^hi^CD127^+^, Lin = CD3, B220, CD11b, Ly6G, TER-119, CD11c, CD5, CD8a, TCRβ, and TCRγδ).

### Cell culture

The MNK-3 cell line (obtained from Guangzhou University of Chinese Medicine) is an effective cell model for studying ILC3s [48]. The cells were cultured in DMEM containing 10% fetal bovine serum and 1% penicillin/streptomycin solution with or without a combination of 25 ng/mL IL-7 (PeproTech), 25 ng/mL IL-23 (R&D), and 10 ng/mL IL-1β (R&D) at 37 °C, 5% CO_2_.

### Cell transfection

MNK3 cells were cultured per well in a 24-well plate in RPMI1640 complete medium supplemented with 10% FBS. The successfully constructed plasmid/siRNA was mixed with the auxiliary transfection reagent lipo2000 to transfect cells for 6 h. After 48 h, the cells were collected for subsequent experiments.

### Western blot analysis

Cells were washed with cold PBS and lysed using RIPA buffer containing phosphatase inhibitors and PMSF (1 mM) on ice. The lysate was then centrifuged at 10,000 × *g* for 10 min at 4 °C to remove insoluble components. The protein concentration of whole cell lysates was measured using the BCA Protein Assay Kit (Beyotime, China). Next, proteins were electrophoresed on SDS-PAGE gels and transferred to a PVDF membrane (Millipore, Bedford, MA, USA) using a wet transfer system at 100 V for 80 min. After blocking for 1 h in 5% non-fat milk, the membrane was incubated with the corresponding primary antibodies overnight at 4 °C, and further incubated with the appropriate horseradish peroxidase-conjugated secondary antibodies for 1 h. Subsequently, the proteins were visualized using chemiluminescent HRP Substrate (Millipore, Billerica, MA, USA) by ChemiDocTM XRS+ (Bio-Rad, Hercules, CA, USA). The antibodies used in this study are listed in Supporting Information Table S2.

### Histopathology analysis

Mouse intestine tissues were dissected and immediately fixed in 4% paraformaldehyde. Paraffin-embedded sections were stained with H&E. Histological scores of colon tissue were evaluated using a previously described scoring criteria [72].

### Cell viability assay

Cells were cultured with the indicated inhibitors for 24 h: zVAD/ Nec-/ Fer-1, or DMSO. Then, cell viability was measured using the alamarBlue Cell Viability Reagent (Invitrogen). All procedures were performed according to the manufacturer’s instructions.

### Lipid ROS production analysis

Analysis of lipid ROS production was performed according to Cao et al. [73]. Briefly, cells were resuspended in 500 μL of PBS containing 2 μM C11-BODIPY (581/591) (#D3861, Invitrogen) and incubated for 30 min at 37 °C in a culture incubator. Then, the cells were washed with fresh PBS and analyzed in the 525/40 nm channel (488 nm laser for excitation) using a flow cytometer for data acquisition. Data were analyzed using the FlowJo V10.0.8 software.

### Induction of ferroptosis

A total of 5 × 10^4^ ILC3s were cultured per well in 96-well U-bottom plates in 200 μL of RPMI1640 complete medium supplemented with 10% FBS and 1× penicillin-streptomycin. Cells were stimulated with IL-1β (10 ng/mL), IL-23 (10 ng/mL), and IL-7(10 ng/mL), treated with vehicle (DMSO) or RSL3 (2–8 μM) / erastin (1–4 μM) with indicated concentrations for 16 /36 h to induce cell ferroptosis, at 37 °C and 5% CO_2_. Then, the cells were washed and collected for subsequent analysis.

### Smart-RNA-seq

To investigate the transcriptome of intestinal ILC3s from Lcn2^fl/fl^ and Rorc^cre^Lcn2^fl/fl^ mice, LPMCs were first isolated by digesting intestinal tissue, followed by flow cytometry sorting to obtain purified ILC3s for subsequent smart-RNA-sequencing analysis. The methods for the library construction and analysis of sequencing results were authored by Genedenovo Biotechnology Co., Ltd (Guangzhou, China).

### Statistical analysis

All experimental data were analyzed using GraphPad Prism (version 8.0; GraphPad Software Inc., San Diego, CA, USA). All statistical analyses were conducted by unpaired two-tailed Student’s *t-*test, non-parametric Mann–Whitney *U* test, or one-way ANOVA test depending on the type of experiments. The statistical significances of differences (*P < 0.05, **P < 0.01, ***P < 0.001, ****P < 0.0001) are indicated in the figures and legends.

## Supplementary information

Supplementary information

Full and uncropped western blots

## Data Availability

The authors declare that the data are present in the paper and/or the supplementary information. Smart-RNA-seq data are available from the Sequence Read Archive (SRA) under accession number PRJNA1149190.

## References

[1] Zhou W, Sonnenberg GF. Activation and Suppression of Group 3 Innate Lymphoid Cells in the Gut. Trends Immunol 2020;41:721–33. 32646594 10.1016/j.it.2020.06.009 PMC7395873

[2] Morita H, Moro K, Koyasu S. Innate lymphoid cells in allergic and nonallergic inflammation. J Allergy Clin Immunol 2016;138:1253–64. 27817797 10.1016/j.jaci.2016.09.011

[3] Ebihara T, Song C, Ryu SH, Plougastel-Douglas B, Yang L, Levanon D, et al. Runx3 specifies lineage commitment of innate lymphoid cells. Nat Immunol 2015;16:1124–33. 26414766 10.1038/ni.3272 PMC4618046

[4] Vivier E, Artis D, Colonna M, Diefenbach A, Di Santo JP, Eberl G, et al. Innate Lymphoid Cells: 10 Years On. Cell 2018;174:1054–66. 30142344 10.1016/j.cell.2018.07.017

[5] Zeng B, Shi S, Ashworth G, Dong C, Liu J, Xing F. ILC3 function as a double-edged sword in inflammatory bowel diseases. Cell Death Dis 2019;10:315. 30962426 10.1038/s41419-019-1540-2 PMC6453898

[6] Forkel M, Mjösberg J. Dysregulation of Group 3 Innate Lymphoid Cells in the Pathogenesis of Inflammatory Bowel Disease. Curr Allergy Asthma Rep. 2016;16:73. 27645534 10.1007/s11882-016-0652-3 PMC5028403

[7] Melo-Gonzalez F, Hepworth MR. Functional and phenotypic heterogeneity of group 3 innate lymphoid cells. Immunology 2017;150:265–75. 27935637 10.1111/imm.12697 PMC5290240

[8] Zhong C, Zheng M, Zhu J. Lymphoid tissue inducer-A divergent member of the ILC family. Cytokine Growth Factor Rev 2018;42:5–12. 29454785 10.1016/j.cytogfr.2018.02.004 PMC6089680

[9] Klose CS, Kiss EA, Schwierzeck V, Ebert K, Hoyler T, d’Hargues Y, et al. A T-bet gradient controls the fate and function of CCR6-RORγt+ innate lymphoid cells. Nature 2013;494:261–65. 23334414 10.1038/nature11813

[10] Guo X, Qiu J, Tu T, Yang X, Deng L, Anders RA, et al. Induction of innate lymphoid cell-derived interleukin-22 by the transcription factor STAT3 mediates protection against intestinal infection. Immunity 2014;40:25–39. 24412612 10.1016/j.immuni.2013.10.021 PMC3919552

[11] Atreya I, Kindermann M, Wirtz S. Innate lymphoid cells in intestinal cancer development. Semin Immunol 2019;41:101267. 30772139 10.1016/j.smim.2019.02.001

[12] Schroeder JH, Howard JK, Lord GM. Transcription factor-driven regulation of ILC1 and ILC3. Trends Immunol 2022;43:564–79. 35618586 10.1016/j.it.2022.04.009 PMC10166716

[13] Keir M, Yi Y, Lu T, Ghilardi N. The role of IL-22 in intestinal health and disease. J Exp Med 2020;217:e20192195. 32997932 10.1084/jem.20192195 PMC7062536

[14] Bernink JH, Krabbendam L, Germar K, de Jong E, Gronke K, Kofoed-Nielsen M, et al. Interleukin-12 and -23 Control Plasticity of CD127(+) Group 1 and Group 3 Innate Lymphoid Cells in the Intestinal Lamina Propria. Immunity 2015;43:146–60. 26187413 10.1016/j.immuni.2015.06.019

[15] Goc J, Lv M, Bessman NJ, Flamar AL, Sahota S, Suzuki H, et al. Dysregulation of ILC3s unleashes progression and immunotherapy resistance in colon cancer. Cell 2021;184:5015–30.e5016. 34407392 10.1016/j.cell.2021.07.029 PMC8454863

[16] Geremia A, Arancibia-Cárcamo CV, Fleming MP, Rust N, Singh B, Mortensen NJ, et al. IL-23-responsive innate lymphoid cells are increased in inflammatory bowel disease. J Exp Med 2011;208:1127–33. 21576383 10.1084/jem.20101712 PMC3173242

[17] Longman RS, Diehl GE, Victorio DA, Huh JR, Galan C, Miraldi ER, et al. CX_3_CR1^+^ mononuclear phagocytes support colitis-associated innate lymphoid cell production of IL-22. J Exp Med 2014;211:1571–83. 25024136 10.1084/jem.20140678 PMC4113938

[18] Boal Carvalho P, Cotter J. Mucosal Healing in Ulcerative Colitis: A Comprehensive Review. Drugs 2017;77:159–73. 28078646 10.1007/s40265-016-0676-y

[19] Kobayashi T, Siegmund B, Le Berre C, Wei SC, Ferrante M, Shen B, et al. Ulcerative colitis. Nat Rev Dis Prim 2020;6:74. 32913180 10.1038/s41572-020-0205-x

[20] Wang X, Huang S, Zhang M, Su Y, Pan Z, Liang J, et al. Gegen Qinlian decoction activates AhR/IL-22 to repair intestinal barrier by modulating gut microbiota-related tryptophan metabolism in ulcerative colitis mice. J Ethnopharmacol 2023;302:115919. 36356716 10.1016/j.jep.2022.115919

[21] Yao F, Deng Y, Zhao Y, Mei Y, Zhang Y, Liu X, et al. A targetable LIFR-NF-κB-LCN2 axis controls liver tumorigenesis and vulnerability to ferroptosis. Nat Commun 2021;12:7333. 34921145 10.1038/s41467-021-27452-9 PMC8683481

[22] Dixon SJ, Lemberg KM, Lamprecht MR, Skouta R, Zaitsev EM, Gleason CE, et al. Ferroptosis: an iron-dependent form of nonapoptotic cell death. Cell 2012;149:1060–72. 22632970 10.1016/j.cell.2012.03.042 PMC3367386

[23] Xie Y, Hou W, Song X, Yu Y, Huang J, Sun X, et al. Ferroptosis: process and function. Cell Death Differ 2016;23:369–79. 26794443 10.1038/cdd.2015.158 PMC5072448

[24] Sun Y, Chen P, Zhai B, Zhang M, Xiang Y, Fang J, et al. The emerging role of ferroptosis in inflammation. Biomed Pharmacother 2020;127:110108. 32234642 10.1016/j.biopha.2020.110108

[25] Lai B, Wu CH, Wu CY, Luo SF, Lai JH. Ferroptosis and Autoimmune Diseases. Front Immunol 2022;13:916664. 35720308 10.3389/fimmu.2022.916664 PMC9203688

[26] Li P, Jiang M, Li K, Li H, Zhou Y, Xiao X, et al. Glutathione peroxidase 4-regulated neutrophil ferroptosis induces systemic autoimmunity. Nat Immunol 2021;22:1107–17. 34385713 10.1038/s41590-021-00993-3 PMC8609402

[27] Yang Y, Wang Y, Guo L, Gao W, Tang TL, Yan M. Interaction between macrophages and ferroptosis. Cell Death Dis 2022;13:355. 35429990 10.1038/s41419-022-04775-z PMC9013379

[28] Cui JX, Xu XH, He T, Liu JJ, Xie TY, Tian W, et al. L-kynurenine induces NK cell loss in gastric cancer microenvironment via promoting ferroptosis. J Exp Clin Cancer Res 2023;42:52. 36855135 10.1186/s13046-023-02629-w PMC9976385

[29] Xu C, Sun S, Johnson T, Qi R, Zhang S, Zhang J, et al. The glutathione peroxidase Gpx4 prevents lipid peroxidation and ferroptosis to sustain Treg cell activation and suppression of antitumor immunity. Cell Rep. 2021;35:109235. 34133924 10.1016/j.celrep.2021.109235

[30] Yang F, Chen Y, Xiao Y, Jiang H, Jiang Z, Yang M, et al. pH-sensitive molybdenum (Mo)-based polyoxometalate nanoclusters have therapeutic efficacy in inflammatory bowel disease by counteracting ferroptosis. Pharm Res 2023;188:106645. 10.1016/j.phrs.2023.106645 36610695

[31] Minaiyan M, Mostaghel E, Mahzouni P. Preventive Therapy of Experimental Colitis with Selected iron Chelators and Anti-oxidants. Int J Prev Med 2012;3:S162–69. 22826760 PMC3399289

[32] Xu J, Liu S, Cui Z, Wang X, Ning T, Wang T, et al. Ferrostatin-1 alleviated TNBS induced colitis via the inhibition of ferroptosis. Biochem Biophys Res Commun 2021;573:48–54. 34388454 10.1016/j.bbrc.2021.08.018

[33] Wang S, Liu W, Wang J, Bai X. Curculigoside inhibits ferroptosis in ulcerative colitis through the induction of GPX4. Life Sci 2020;259:118356. 32861798 10.1016/j.lfs.2020.118356

[34] Xu M, Tao J, Yang Y, Tan S, Liu H, Jiang J, et al. Ferroptosis involves in intestinal epithelial cell death in ulcerative colitis. Cell Death Dis 2020;11:86. 32015337 10.1038/s41419-020-2299-1 PMC6997394

[35] Huang F, Zhang S, Li X, Huang Y, He S, Luo L. STAT3-mediated ferroptosis is involved in ulcerative colitis. Free Radic Biol Med 2022;188:375–85. 35779691 10.1016/j.freeradbiomed.2022.06.242

[36] Zhang X, Ma Y, Lv G, Wang H. Ferroptosis as a therapeutic target for inflammation-related intestinal diseases. Front Pharmacol 2023;14:1095366. 36713828 10.3389/fphar.2023.1095366 PMC9880170

[37] He F, Zhang P, Liu J, Wang R, Kaufman RJ, Yaden BC, et al. ATF4 suppresses hepatocarcinogenesis by inducing SLC7A11 (xCT) to block stress-related ferroptosis. J Hepatol 2023;79:362–77. 36996941 10.1016/j.jhep.2023.03.016 PMC11332364

[38] Ye P, Mimura J, Okada T, Sato H, Liu T, Maruyama A, et al. Nrf2- and ATF4-dependent upregulation of xCT modulates the sensitivity of T24 bladder carcinoma cells to proteasome inhibition. Mol Cell Biol 2014;34:3421–34. 25002527 10.1128/MCB.00221-14 PMC4135628

[39] Lim JKM, Delaidelli A, Minaker SW, Zhang HF, Colovic M, Yang H, et al. Cystine/glutamate antiporter xCT (SLC7A11) facilitates oncogenic RAS transformation by preserving intracellular redox balance. Proc Natl Acad Sci Usa 2019;116:9433–42. 31000598 10.1073/pnas.1821323116 PMC6511045

[40] Habib E, Linher-Melville K, Lin HX, Singh G. Expression of xCT and activity of system xc(-) are regulated by NRF2 in human breast cancer cells in response to oxidative stress. Redox Biol 2015;5:33–42. 25827424 10.1016/j.redox.2015.03.003 PMC4392061

[41] Jiang L, Kon N, Li T, Wang SJ, Su T, Hibshoosh H, et al. Ferroptosis as a p53-mediated activity during tumour suppression. Nature 2015;520:57–62. 25799988 10.1038/nature14344 PMC4455927

[42] Yang WS, SriRamaratnam R, Welsch ME, Shimada K, Skouta R, Viswanathan VS, et al. Regulation of ferroptotic cancer cell death by GPX4. Cell 2014;156:317–31. 24439385 10.1016/j.cell.2013.12.010 PMC4076414

[43] Smillie CS, Biton M, Ordovas-Montanes J, Sullivan KM, Burgin G, Graham DB, et al. Intra- and Inter-cellular Rewiring of the Human Colon during Ulcerative Colitis. Cell 2019;178:714–30.e722. 31348891 10.1016/j.cell.2019.06.029 PMC6662628

[44] Skouta R, Dixon SJ, Wang J, Dunn DE, Orman M, Shimada K, et al. Ferrostatins inhibit oxidative lipid damage and cell death in diverse disease models. J Am Chem Soc 2014;136:4551–56. 24592866 10.1021/ja411006a PMC3985476

[45] Kim R, Hashimoto A, Markosyan N, Tyurin VA, Tyurina YY, Kar G, et al. Ferroptosis of tumour neutrophils causes immune suppression in cancer. Nature 2022;612:338–46. 36385526 10.1038/s41586-022-05443-0 PMC9875862

[46] Aujla SJ, Chan YR, Zheng M, Fei M, Askew DJ, Pociask DA, et al. IL-22 mediates mucosal host defense against Gram-negative bacterial pneumonia. Nat Med 2008;14:275–81. 18264110 10.1038/nm1710 PMC2901867

[47] Koroleva EP, Halperin S, Gubernatorova EO, Macho-Fernandez E, Spencer CM, Tumanov AV. Citrobacter rodentium-induced colitis: A robust model to study mucosal immune responses in the gut. J Immunol Methods 2015;421:61–72. 25702536 10.1016/j.jim.2015.02.003 PMC12955730

[48] Allan DS, Kirkham CL, Aguilar OA, Qu LC, Chen P, Fine JH, et al. An in vitro model of innate lymphoid cell function and differentiation. Mucosal Immunol 2015;8:340–51. 25138665 10.1038/mi.2014.71

[49] Sefik E, Geva-Zatorsky N, Oh S, Konnikova L, Zemmour D, McGuire AM, et al. MUCOSAL IMMUNOLOGY. Individual intestinal symbionts induce a distinct population of RORγ^+^ regulatory T cells. Science 2015;349:993–97. 26272906 10.1126/science.aaa9420 PMC4700932

[50] Eberl G. RORγt, a multitask nuclear receptor at mucosal surfaces. Mucosal Immunol 2017;10:27–34. 27706126 10.1038/mi.2016.86

[51] Lyu M, Suzuki H, Kang L, Gaspal F, Zhou W, Goc J, et al. ILC3s select microbiota-specific regulatory T cells to establish tolerance in the gut. Nature 2022;610:744–51. 36071169 10.1038/s41586-022-05141-x PMC9613541

[52] Jarade A, Di Santo JP, Serafini N. Group 3 innate lymphoid cells mediate host defense against attaching and effacing pathogens. Curr Opin Microbiol 2021;63:83–91. 34274597 10.1016/j.mib.2021.06.005

[53] Seshadri S, Allan DSJ, Carlyle JR, Zenewicz LA. Bacillus anthracis lethal toxin negatively modulates ILC3 function through perturbation of IL-23-mediated MAPK signaling. PLoS Pathog 2017;13:e1006690. 29059238 10.1371/journal.ppat.1006690 PMC5695638

[54] Chakraborty S, Kaur S, Guha S, Batra SK. The multifaceted roles of neutrophil gelatinase associated lipocalin (NGAL) in inflammation and cancer. Biochim Biophys Acta 2012;1826:129–69. 22513004 10.1016/j.bbcan.2012.03.008 PMC3362670

[55] Jaberi SA, Cohen A, D’Souza C, Abdulrazzaq YM, Ojha S, Bastaki S, et al. Lipocalin-2: Structure, function, distribution and role in metabolic disorders. Biomed Pharmacother 2021;142:112002. 34463264 10.1016/j.biopha.2021.112002

[56] Xiao X, Yeoh BS, Vijay-Kumar M. Lipocalin 2: An Emerging Player in Iron Homeostasis and Inflammation. Annu Rev Nutr 2017;37:103–30. 28628361 10.1146/annurev-nutr-071816-064559

[57] Deng L, He S, Li Y, Ding R, Li X, Guo N, et al. Identification of Lipocalin 2 as a Potential Ferroptosis-related Gene in Ulcerative Colitis. Inflamm Bowel Dis 2023;29:1446–57. 37000707 10.1093/ibd/izad050

[58] Monteleone I, Marafini I, Dinallo V, Di Fusco D, Troncone E, Zorzi F, et al. Sodium chloride-enriched Diet Enhanced Inflammatory Cytokine Production and Exacerbated Experimental Colitis in Mice. J Crohns Colitis 2017;11:237–45. 27473029 10.1093/ecco-jcc/jjw139

[59] Huang J, Zhang J, Ma J, Ma J, Liu J, Wang F, et al. Inhibiting Ferroptosis: A Novel Approach for Ulcerative Colitis Therapeutics. Oxid Med Cell Longev 2022;2022:9678625. 35378823 10.1155/2022/9678625 PMC8976662

[60] Ocansey DKW, Yuan J, Wei Z, Mao F, Zhang Z. Role of ferroptosis in the pathogenesis and as a therapeutic target of inflammatory bowel disease (Review). Int J Mol Med 2023;51:53. 37203397 10.3892/ijmm.2023.5256 PMC10198063

[61] Chen Y, Zhang P, Chen W, Chen G. Ferroptosis mediated DSS-induced ulcerative colitis associated with Nrf2/HO-1 signaling pathway. Immunol Lett 2020;225:9–15. 32540488 10.1016/j.imlet.2020.06.005

[62] Ye Y, Liu L, Feng Z, Liu Y, Miao J, Wei X, et al. The ERK-cPLA2-ACSL4 axis mediating M2 macrophages ferroptosis impedes mucosal healing in ulcerative colitis. Free Radic Biol Med 2024;214:219–35. 38367927 10.1016/j.freeradbiomed.2024.02.016

[63] Rankin LC, Girard-Madoux MJ, Seillet C, Mielke LA, Kerdiles Y, Fenis A, et al. Complementarity and redundancy of IL-22-producing innate lymphoid cells. Nat Immunol 2016;17:179–86. 26595889 10.1038/ni.3332 PMC4720992

[64] Bank U, Deiser K, Plaza-Sirvent C, Osbelt L, Witte A, Knop L, et al. c-FLIP is crucial for IL-7/IL-15-dependent NKp46(+) ILC development and protection from intestinal inflammation in mice. Nat Commun 2020;11:1056. 32103006 10.1038/s41467-020-14782-3 PMC7044440

[65] Chen H, Qian Y, Jiang C, Tang L, Yu J, Zhang L, et al. Butyrate ameliorated ferroptosis in ulcerative colitis through modulating Nrf2/GPX4 signal pathway and improving intestinal barrier. Biochim Biophys Acta Mol Basis Dis 2024;1870:166984. 38061600 10.1016/j.bbadis.2023.166984

[66] Colonna M. Innate Lymphoid Cells: Diversity, Plasticity, and Unique Functions in Immunity. Immunity 2018;48:1104–17. 29924976 10.1016/j.immuni.2018.05.013 PMC6344351

[67] Coorens M, Rao A, Gräfe SK, Unelius D, Lindforss U, Agerberth B, et al. Innate lymphoid cell type 3-derived interleukin-22 boosts lipocalin-2 production in intestinal epithelial cells via synergy between STAT3 and NF-κB. J Biol Chem 2019;294:6027–41. 30782844 10.1074/jbc.RA118.007290 PMC6463718

[68] Xu HM, Huang HL, Xu J, He J, Zhao C, Peng Y, et al. Cross-Talk Between Butyric Acid and Gut Microbiota in Ulcerative Colitis Following Fecal Microbiota Transplantation. Front Microbiol 2021;12:658292. 33912150 10.3389/fmicb.2021.658292 PMC8071877

[69] Li W, Zhang L, Xu Q, Yang W, Zhao J, Ren Y, et al. Taxifolin Alleviates DSS-Induced Ulcerative Colitis by Acting on Gut Microbiome to Produce Butyric Acid. Nutrients 2022;14:1069. 35268045 10.3390/nu14051069 PMC8912346

[70] Deng F, Zhao BC, Yang X, Lin ZB, Sun QS, Wang YF, et al. The gut microbiota metabolite capsiate promotes Gpx4 expression by activating TRPV1 to inhibit intestinal ischemia reperfusion-induced ferroptosis. Gut Microbes 2021;13:1–21. 33779497 10.1080/19490976.2021.1902719 PMC8009132

[71] Liu S, Gao Z, He W, Wu Y, Liu J, Zhang S, et al. The gut microbiota metabolite glycochenodeoxycholate activates TFR-ACSL4-mediated ferroptosis to promote the development of environmental toxin-linked MAFLD. Free Radic Biol Med 2022;193:213–26. 36265794 10.1016/j.freeradbiomed.2022.10.270

[72] Huang J, Lee HY, Zhao X, Han J, Su Y, Sun Q, et al. Interleukin-17D regulates group 3 innate lymphoid cell function through its receptor CD93. Immunity 2021;54:673–86.e674. 33852831 10.1016/j.immuni.2021.03.018

[73] Cao J, Chen X, Jiang L, Lu B, Yuan M, Zhu D, et al. DJ-1 suppresses ferroptosis through preserving the activity of S-adenosyl homocysteine hydrolase. Nat Commun 2020;11:1251. 32144268 10.1038/s41467-020-15109-y PMC7060199

[74] Linkermann A, Skouta R, Himmerkus N, Mulay SR, Dewitz C, De Zen F, et al. Synchronized renal tubular cell death involves ferroptosis. Proc Natl Acad Sci Usa 2014;111:16836–41. 25385600 10.1073/pnas.1415518111 PMC4250130

[75] Tonnus W, Meyer C, Steinebach C, Belavgeni A, von Mässenhausen A, Gonzalez NZ, et al. Dysfunction of the key ferroptosis-surveilling systems hypersensitizes mice to tubular necrosis during acute kidney injury. Nat Commun 2021;12:4402. 34285231 10.1038/s41467-021-24712-6 PMC8292346

