# Supplementary material for: GPX4 restricts ferroptosis of NKp46+ILC3s to control intestinal inflammation
Source: Cell Death Dis. 2024 Sep 19;15(9):687. doi: 10.1038/s41419-024-07060-3 (PMC11413021; doi:10.1038/s41419-024-07060-3)
Supplement: Supplementary file 1 — Supplementary information [file 41419_2024_7060_MOESM1_ESM.pdf]

Supplementary information

Supplementary Figure 1-11

Supplementary Table 1- 3

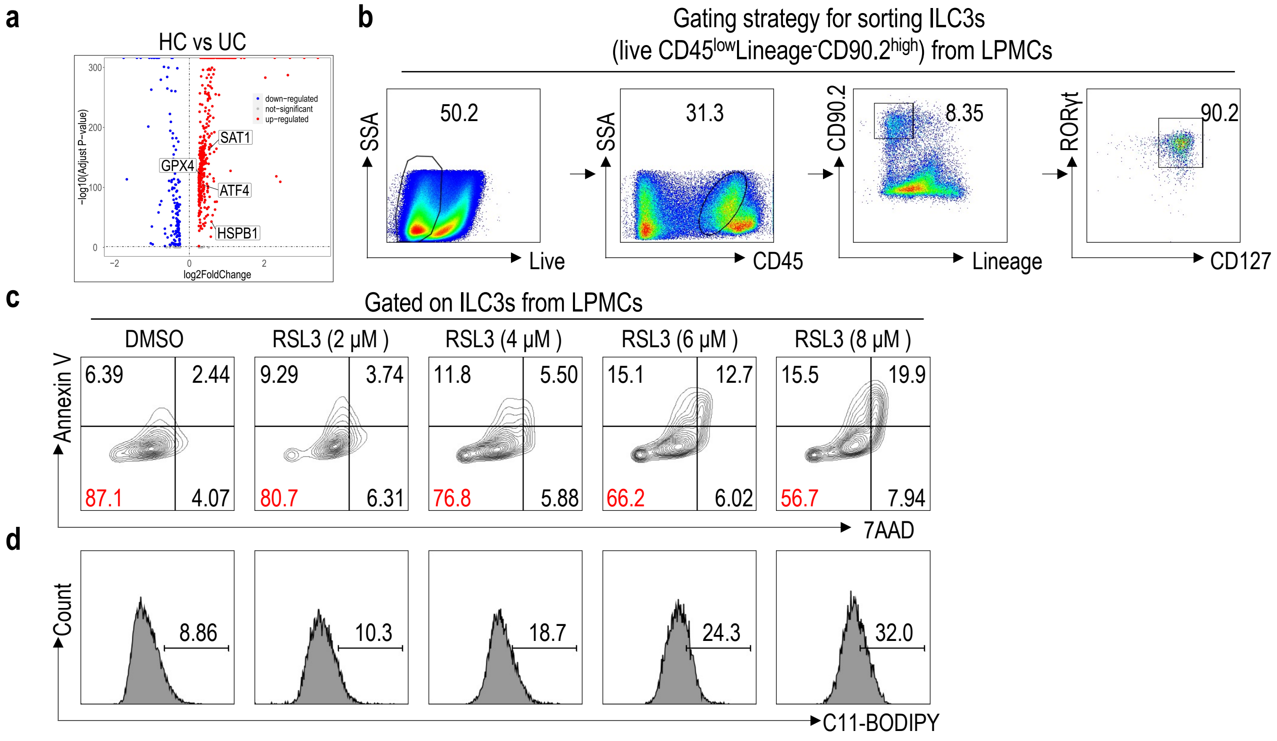

Supplementary Figure 1. ILC3s showed intrinsic susceptibility to ferroptosis. a Volcano plot of differentially expressed genes between ILC3s derived from the mucosa of healthy controls (HC) and patients with UC based on single-cell sequencing data (accession number SCP259) obtained from the Single Cell Portal. The red and blue dots represent genes that are upregulated and downregulated, respectively, in ILC3s of patients with UC relative to HC. **b** Sorting strategy of ILC3s for *in vitro* cultivation isolated from LPMCs of WT mice (gated on live $CD45^{low}Lineage^{-}CD90.2^{high}$). As shown in the figure, most of $CD45^{low}Lineage^{-}CD90.2^{high}$ LPMCs (90.2%) were $ROR\gamma t^{+}$ ILC3s. **c** The representative flow cytometry plots of the percentage of 7-AAD and annexin V double-negative cells and **d** lipid ROS production in sorted ILC3s from the LPMCs after treated with RSL3 (2–8 μM) or vehicle (DMSO). Data are representative of at least three independent experiments.

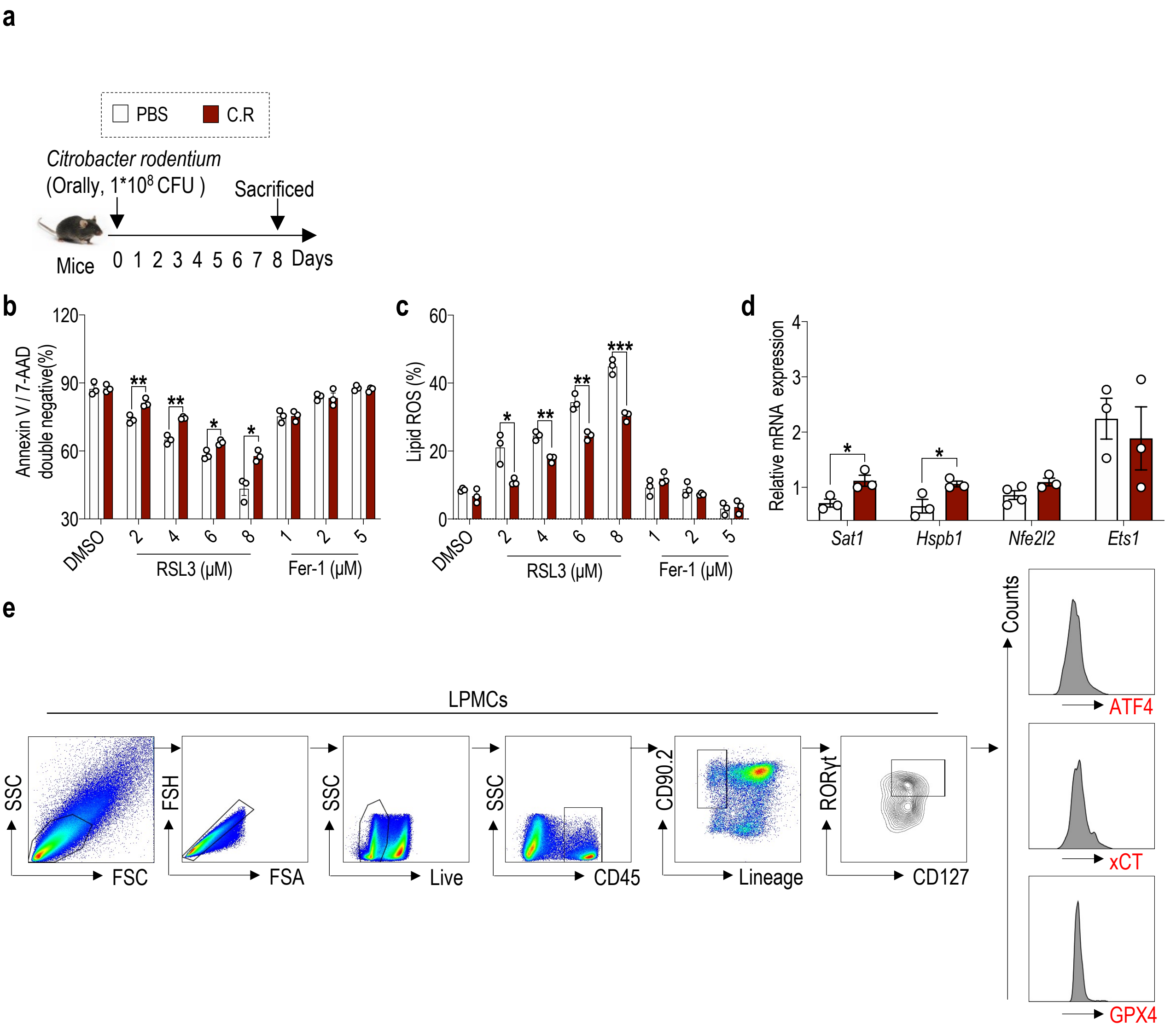

Supplementary Figure 2. The intestinal ILC3s of mice infected with C. rodentium exhibit enhanced resistance to ferroptosis. a WT mice were orally administered with or without (PBS) 1 × 10^8 CFU of C. rodentium (C.R). After 8 days, the intestinal LPMCs were isolated and sorted for ILC3s, followed by treatment with RSL3 (2-8 μM), Fer-1 (1-5 μM), or vehicle (DMSO) for 16 h. **b** The statistical results of the percentage of 7-AAD and annexin V double-negative population and **c** the lipid ROS production are shown. (n=3) **d** The mRNA expression levels of *Sat1*, *Hspb1*, *Nfe2l2*, and *Ets1* in the sorted intestinal ILC3s from the indicated mice were assayed by qRT-PCR. The relative gene expression was normalized to β-actin. (n=3) **e** The gating strategy for the expression of ATF4, xCT, and GPX4 in LPMCs derived ILC3s of mice. Data are representative of at least three independent experiments. Data are presented as the mean±SEM, and statistical significance was determined by two-sided unpaired t-test (**b**–**d**). *P < 0.05; **P < 0.01; ***P < 0.001.

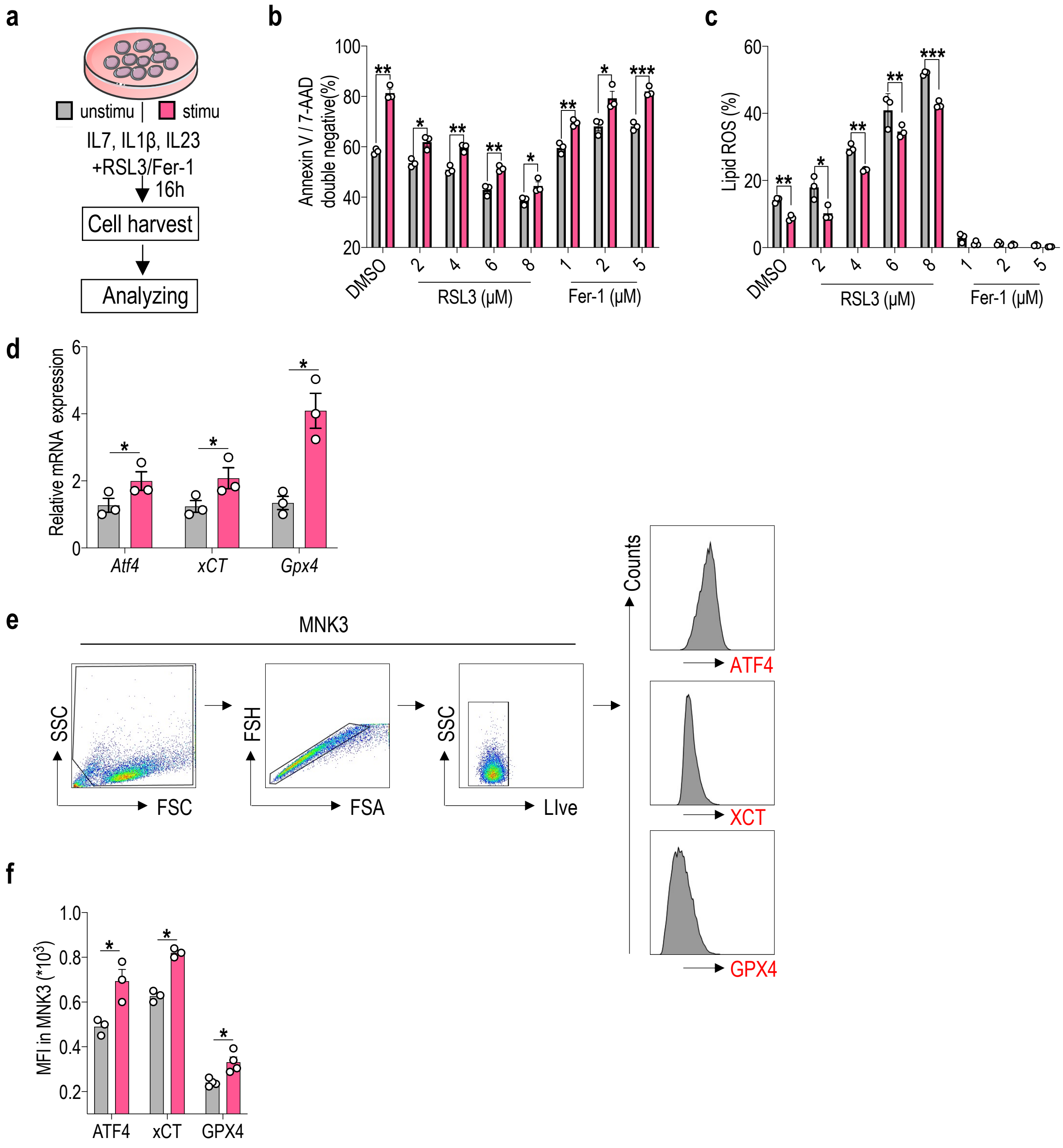

Supplementary Figure 3. The susceptibility of MNK3 cells to ferroptosis is attenuated upon activation. a, MNK3 cells were treated with RSL3 or Fer-1 or vehicle (DMSO), in the presence or absence of IL-7, IL-1β, and IL-23 (100ng/ml) stimulation. After 16 h, the cells were harvested for analysis. The statistical results of **b,** the percentage of 7-AAD and annexin V double-negative population and **c,** the lipid ROS production are shown. (n=3) **d,** Comparative analysis of mRNA expression levels of *Atf4*, *xCT*, and *Gpx4* in unstimulated or stimulated MNK3 cells. The relative gene expression was normalized to β-actin. (n=3) **e,** The gating strategy for the expression of ATF4, xCT, and GPX4 in MNK3 cells. **f,** Comparative analysis of protein expression levels of ATF4, xCT, and GPX4 in unstimulated versus stimulated MNK3 cells. (n=3) Data are representative of at least three independent experiments. Data are presented as the mean±SEM, and statistical significance was determined by two-sided unpaired t-test (**b**–**d, f**). *P < 0.05; **P < 0.01; ***P < 0.001.

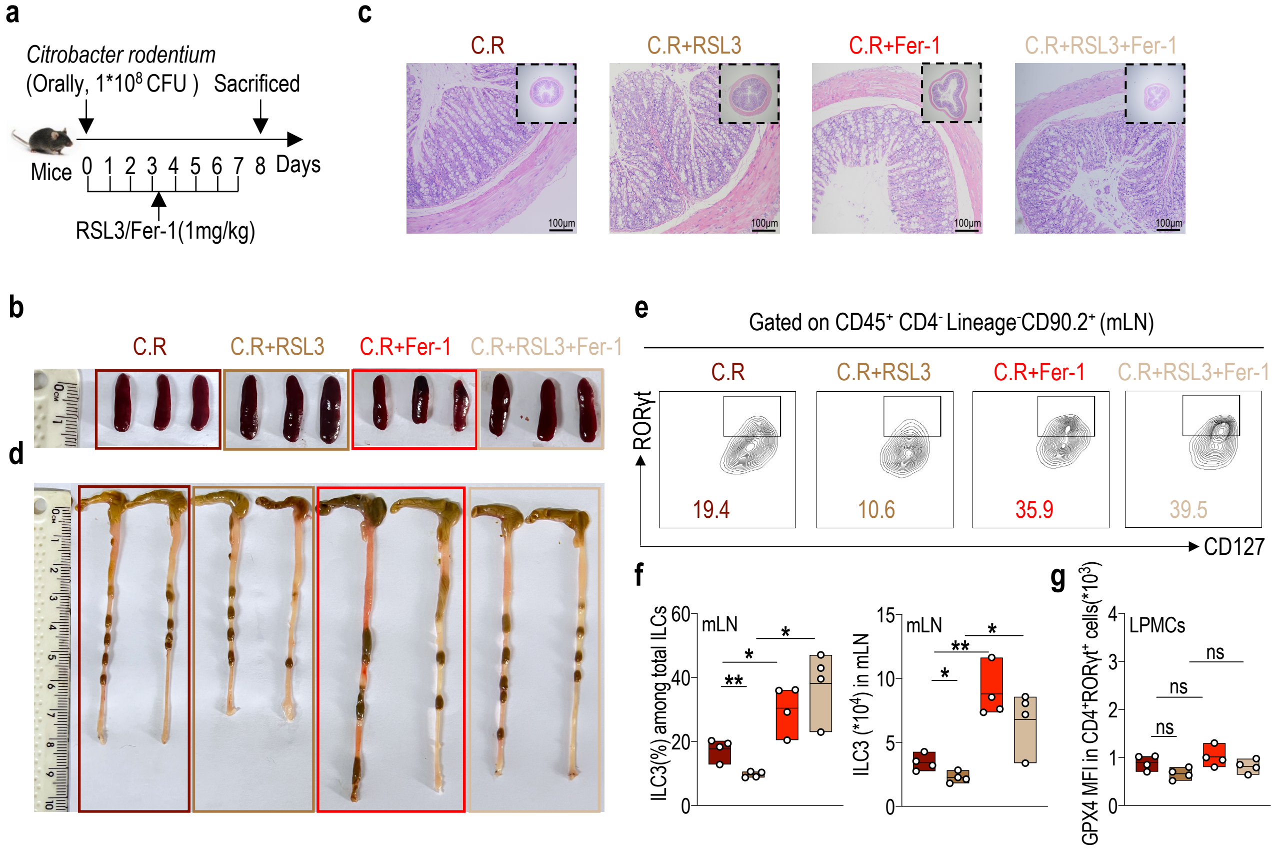

Supplementary Figure 4. Alterations in pathological characteristics and cell phenotype following ferroptosis intervention in C.R-infected mice. a Schematic illustration of the strategy of the experimental setup of C. rodentium (C.R) infected mouse treatment models. Mice infected with C.R were treated with RSL3 (10 mg/kg/day, i.p.) or Fer-1 (1 mg/kg/day, i.p.), either alone or in combination (RSL3, 10 mg/kg/day; Fer-1, 10 mg/kg/day, i.p.), and tissue samples were collected on Day 8. **b** Representative spleen images of the indicated groups of mice are shown. **c** Representative H&E staining of the colon sections from indicated groups of mice with C.R infection (scale bars: 100 μm). **d** Representative colon length images of the indicated groups of mice are shown. **e** The representative flow cytometry plots and **f** statistical results of the proportion (left) and absolute number (right) of ILC3s in mLN of indicated mice. (n=4) **g** The statistical results of the MFI for GPX4 in $CD4^+ROR\gamma t^+$ cells from LPMCs of indicated mice. (n = 4) Data are presented as the median, and statistical significance was determined by one-way ANOVA test (**f** and **g**). *$P < 0.05$; **$P < 0.01$; ***$P < 0.001$.

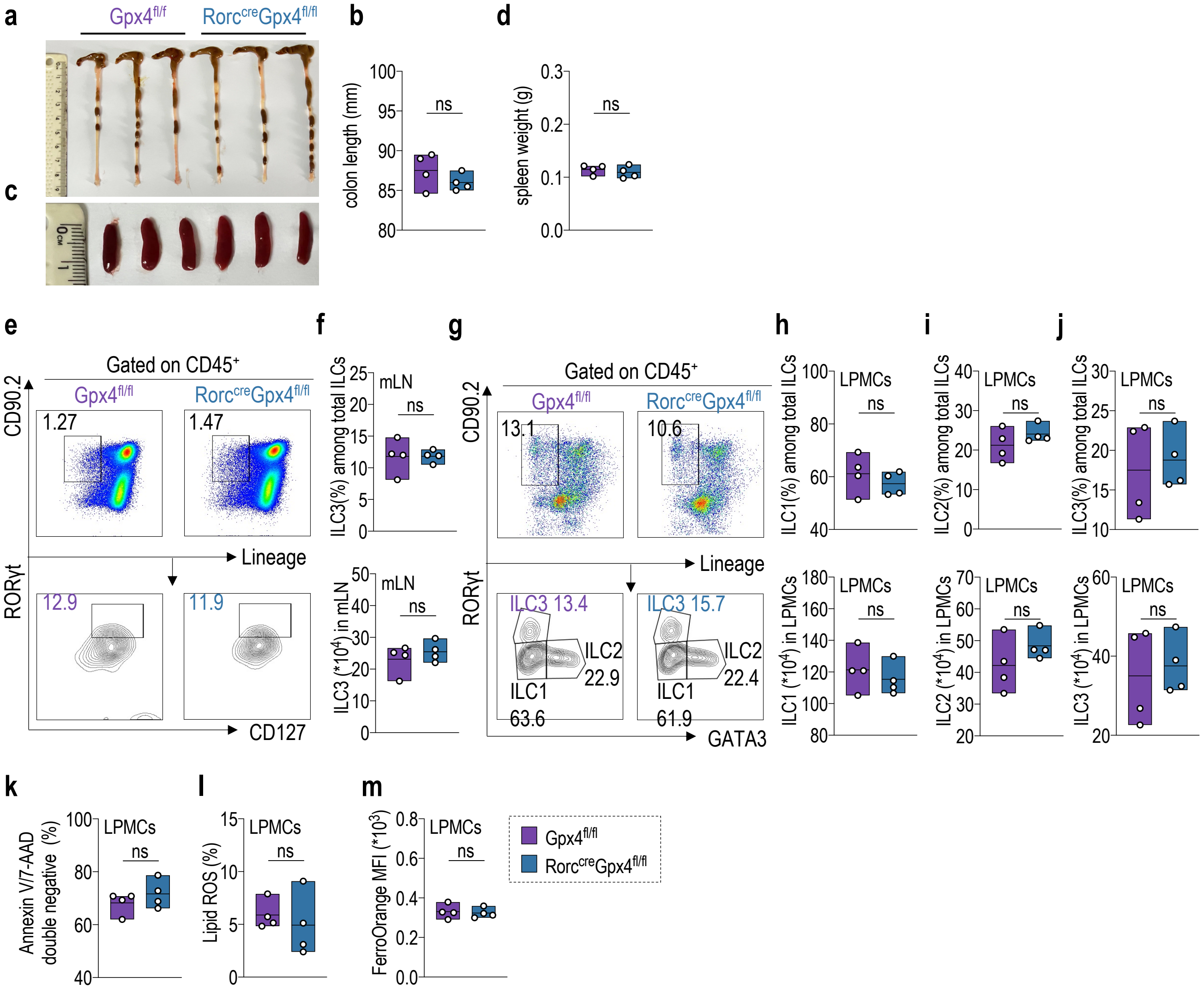

Supplementary Figure 5. Phenotypic and ferroptotic characteristics of intestinal ILC3s in mice following conditional knockout of Gpx4 on Rorc. a and **c,** Representative images and statistical results of **b** colon length and **d** spleen weight from the indicated mice are shown. (n=4) **e** The representative flow cytometry plots and **f** statistical results of the proportion (upper) and absolute number (lower) of ILC3s in mLN of indicated mice. (n=4) **g** The representative flow cytometry plots and statistical results of the proportion (upper) and absolute number (lower) of **h** ILC1s, **i** ILC2s, and **j** ILC3s in LPMCs of indicated mice. (n=4) **k** Statistical results of the percentage of annexin V and 7-AAD double-negative population and **l** lipid ROS production in LPMCs-derived ILC3s from the indicated mice. (n=4) **m** Fe^{2+} level in ILC3s from LPMCs was analyzed by FerroOrange, and the statistical results are shown. (n=4) Data are presented as the median, and statistical significance was determined by two-sided unpaired t-test (**b**, **d**, **f**, **h-m**). *P < 0.05; **P < 0.01; ***P < 0.001.

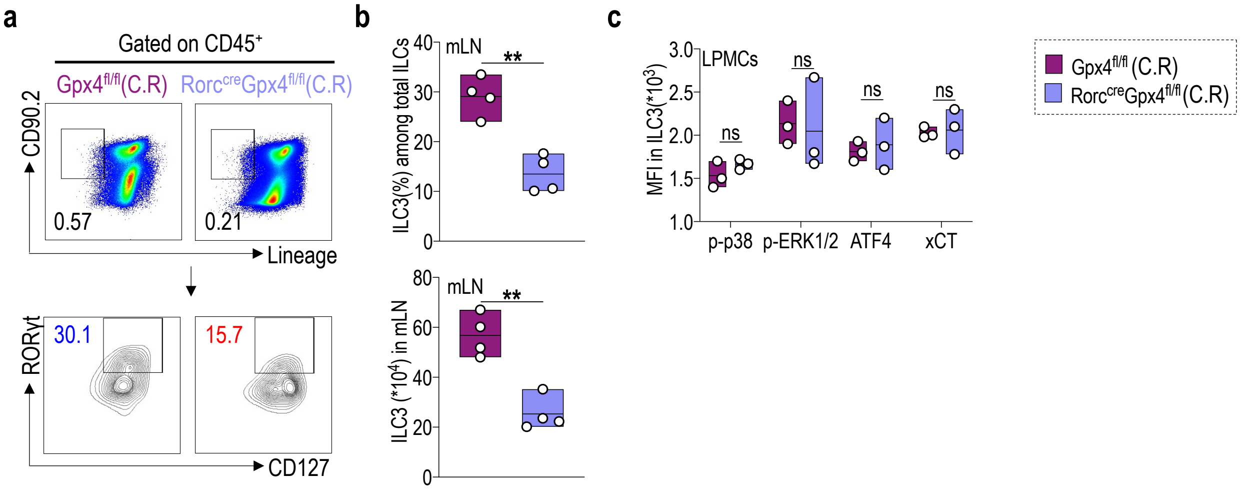

Supplementary Figure 6. Alterations in the number of ILC3s from mLNs and ferroptotic related proteins of Rorc^{cre}Gpx4$^{fl/fl}$ mice infected with C.R. a The representative flow cytometry plots and **b** statistical results of the proportion (upper) and absolute number (lower) of ILC3s in mLN of indicated mice. (n=4) **c** Statistical results of MFI for p-p38-Thr180/Tyr182, p-ERK1/2 (Thr204/Thr187), ATF4 and xCT expression in ILC3s from LPMCs of indicated mice. (n = 3) Data are presented as the median, and statistical significance was determined by two-sided unpaired t-test (**b, c**). *P < 0.05; **P < 0.01; ***P < 0.001.

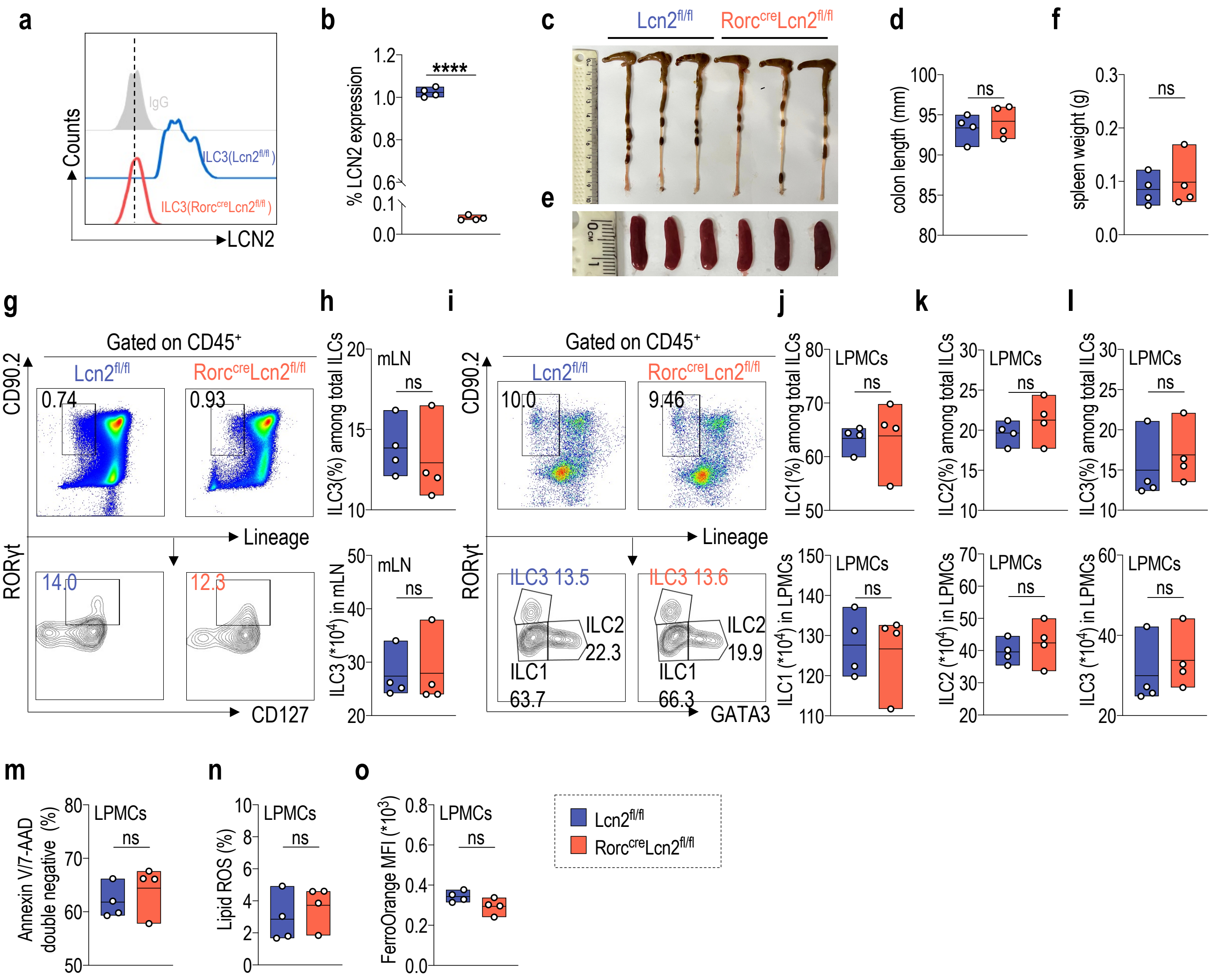

Supplementary Figure 7. No significant differences in the phenotype and ferroptosis characteristics of intestinal ILC3s between Lcn2$^{fl/fl}$ and Rorc^{cre}Lcn2$^{fl/fl}$ mice under physiological conditions. a Representative flow cytometry and **b** statistical analysis of LCN2 expression in intestinal ILC3 cells from Lcn2$^{fl/fl}$ and Lcn2$^{fl/fl}$ Rorc^{cre} mice. (n=4) **c** and **e** Representative images and statistical results of **d** colon length and **f** spleen weight from the indicated mice are shown. (n=4) **g** The representative flow cytometry plots and **h** statistical results of the proportion (upper) and absolute number (lower) of ILC3s in mLN of indicated mice. (n=4) **i** The representative flow cytometry plots and statistical results of the proportion (upper) and absolute number (lower) of **j** ILC1s **k** ILC2s, and **l** ILC3s in LPMCs of indicated mice. (n=4) Statistical results of **m** the percentage of annexin V and 7-AAD double-negative population and **n** lipid ROS production in LPMCs-derived ILC3s from the indicated mice. (n=4) **o** Fe^{2+} level in ILC3s from LPMCs was analyzed by FerroOrange, and the statistical results are shown. (n=4) Data are presented as the median, and statistical significance was determined by two-sided unpaired t-test (**b**, **d**, **f**, **h**, **j-o**). *P < 0.05; **P < 0.01; ***P < 0.001.

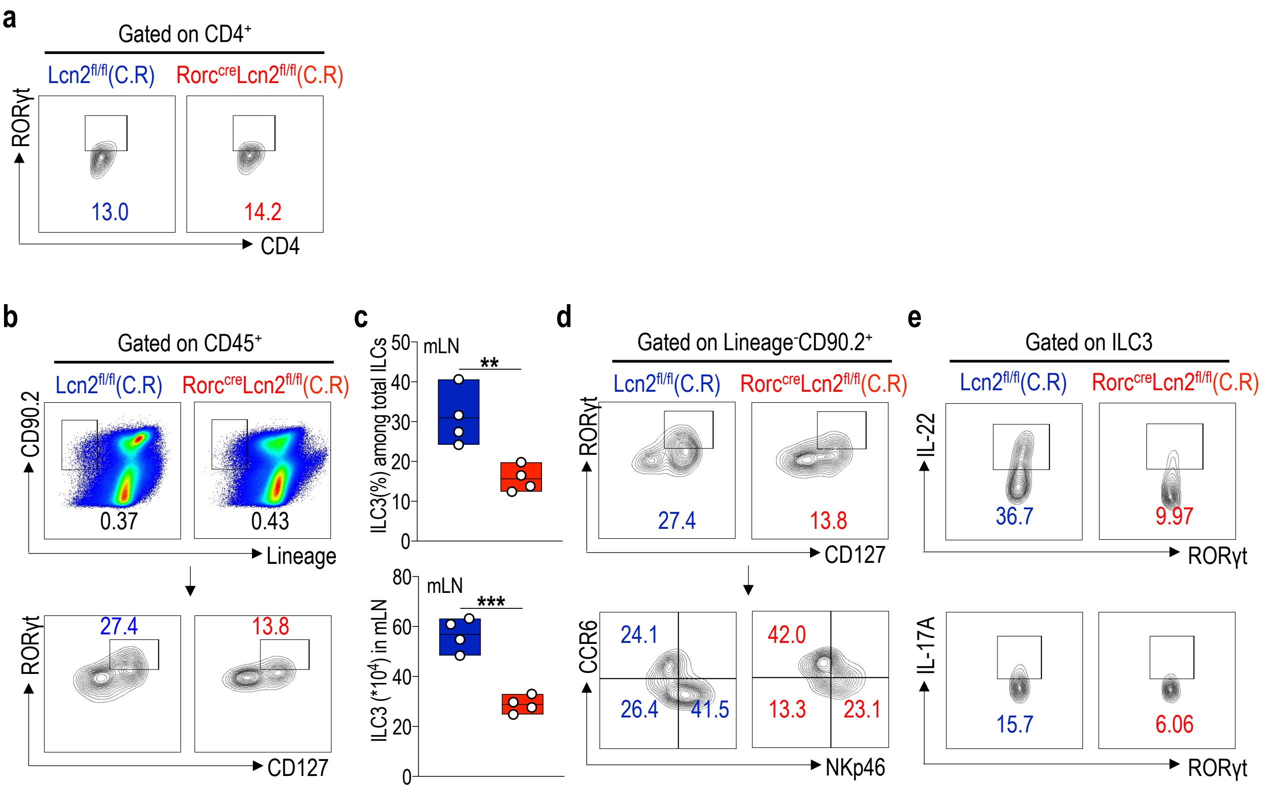

Supplementary Figure 8. Rorc^{cre}Lcn2$^{fl/fl}$ mice infected with C.R exhibit significant alterations in the proportion and functionality of intestinal ILC3s compared to Lcn2$^{fl/fl}$ mice. a The representative flow cytometry plots of intestinal CD4$^+$RORγt$^+$ cells from indicated mice. **b** The representative flow cytometry plots and **c** statistical results of the proportion (upper) and absolute number (lower) of ILC3s in mLN of indicated mice. (n=4) **d** The representative flow cytometry plots of ILC3s, including NKP46$^+$ILC3, CCR6$^+$ILC3, and DN cell subsets in LPMCs of indicated mice. **e** Representative flow cytometry plots of IL-22- (upper), and IL-17A-positive (lower) ILC3s in LPMCs of indicated mice. Data are presented as the median, and statistical significance was determined by two-sided unpaired t-test (**c**). *P < 0.05; **P < 0.01; ***P < 0.001.

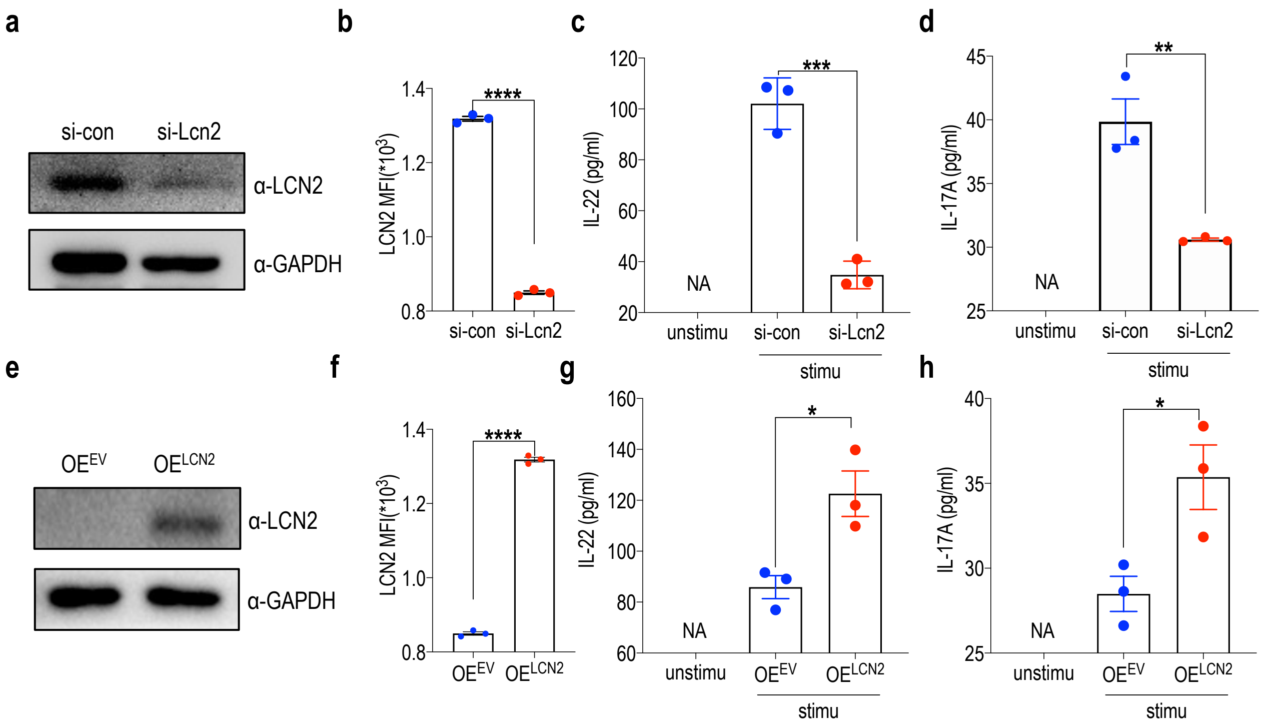

Supplementary Figure 9. Intervening in the expression of LCN2 significantly modulates the functionality of MNK3 cells. a Western blotting of LCN2 expression level in MNK3 cells transfected with si-con or si-Lcn2. **b** Expression levels of LCN2 in indicated MNK3 cells. (n=3) Secreted levels of **c** IL-22 and **d** IL-17A in culture supernatant after transfection with si-con or si-Lcn2 for 48h. (n=3) **e** Western blotting of LCN2 expression level in MNK3 cells transfected with plasmids encoding LCN2 (OE^{LCN2}) or empty vector (OE^{EV}). **f** Expression levels of LCN2 in indicated MNK3 cells. (n=3) Secreted levels of **g** IL-22 and **h** IL-17A in culture supernatant after transfection with plasmids OE^{LCN2} or OE^{EV}. (n=3) Data are presented as the mean±SEM, and statistical significance was determined by two-sided unpaired t-test (**b-d, f-h**). *P < 0.05; **P < 0.01; ***P < 0.001.

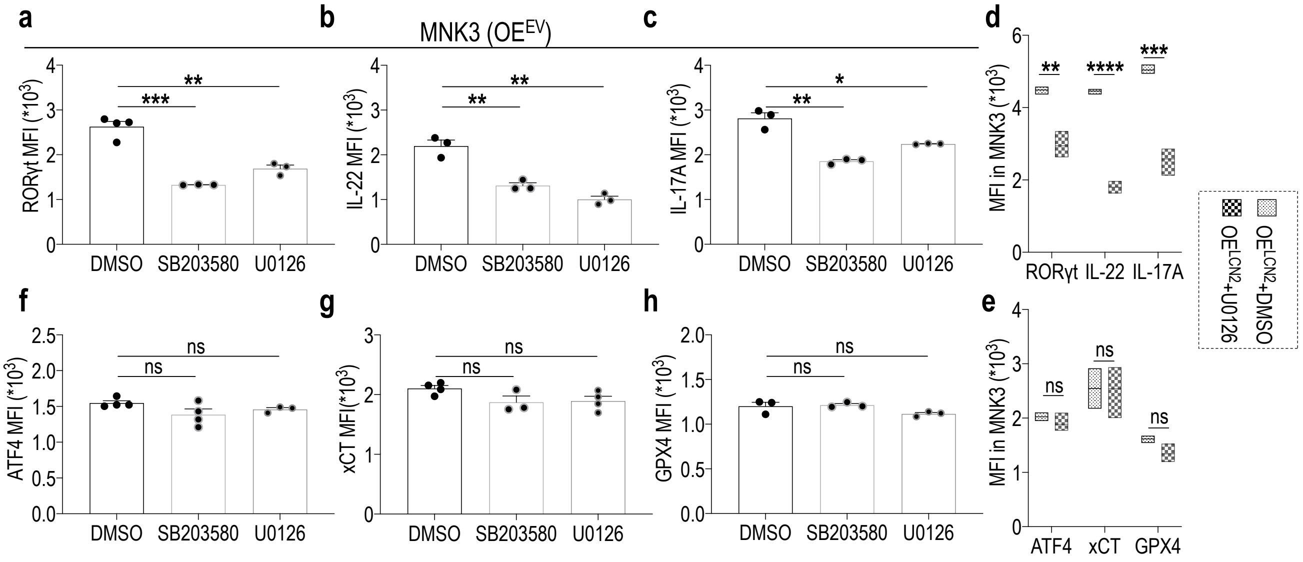

Supplementary Figure 10. The ERK1/2 signaling pathway does not participate in the regulation of the downstream molecular mechanism of LCN2 associated with ferroptosis. After transfecting MNK3 cells with plasmids OE^{LCN2} or OE^{EV}, the cells were exposed to erastin for 16 h in the presence or absence of the p38 inhibitor SB203580 or p-ERK1/2 inhibitor U0126. Expression levels of **a** RORγt, **b** IL-22, and **c** IL-17A in indicated MNK3 cells transfected with plasmids OE^{EV}. (n=3) **d** Expression levels of RORγt, IL-22, and IL-17A in indicated MNK3 cells transfected with plasmids OE^{LCN2}. (n=3) **e** Expression levels of ATF4, xCT, and GPX4 in indicated MNK3 cells transfected with plasmids OE^{LCN2}. (n=3) Expression levels of **f** ATF4, **g** xCT, and **h** GPX4 in indicated MNK3 cells transfected with plasmids OE^{EV}. (n=3) Data are presented as the mean±SEM or median, and statistical significance was determined by two-sided unpaired t-test (**a**-**h**). $*P < 0.05$; $**P < 0.01$; $***P < 0.001$.

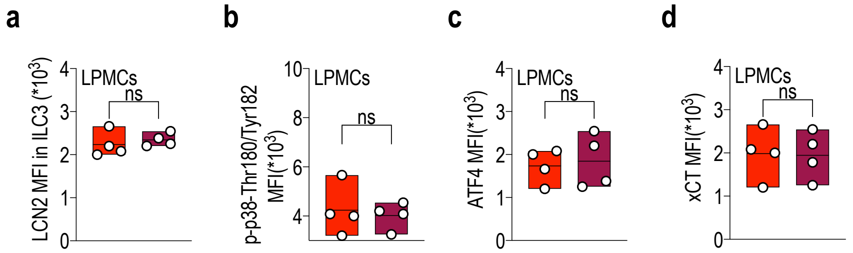

Supplementary Figure 11. The restoration of GPX4 expression in Rorc^{cre}Lcn2$^{fl/fl}$ mice treated with ferroptosis inhibitor have no impact on the LCN2 and its downstream p38-ATF4-xCT axis. Statistical results of MFI for **a** LCN2, **b** p-p38-Thr180/Tyr182, **c** ATF4, and **d** xCT expression in indicated mice. (n=4) Data are presented as the median, and statistical significance was determined by two-sided unpaired t-test (**a**-**d**). *P < 0.05; **P < 0.01; ***P < 0.001.

Supplementary Table 1. Reagents used in this study

Name	Cat#	Vendor	Country
Fix & Perm Solution	554722	BD Biosciences	USA
DMSO	V900090	Sigma	USA
RPMI-1640	01-100-1ACS	BI	Israel
FBS	04-001-1ACS	BI	Israel
Penicillin-Streptomycin Solution	03-031-1B	BI	Israel
DMEM	06-1055-57-1A	BI	Israel
Streptavidin Microbeads	557812	BD Biosciences	USA
Percoll	17-0891-09	GE	Sweden
DTT	P001008	Amresco	USA
Collagenase I	17104019	Gibco	USA
Hyaluronidase	H3506-1G	SIGMA	USA
DNase I	B002138-0025	Sangon Biotech	China
RIPA Lysis Buffer	P0013B	Beyotime	China
Bicinchoninic Acid Protein Assay Kit	P0012S	Beyotime	China
30% Acrylamide/Methylene Bisacrylamide	B546017-0500	Sangon Biotech	China
Tris-HCl-SDS PH=6.8	C526034-0250	Sangon Biotech	China
Tris-HCl-SDS PH=8.8	C526033-0250	Sangon Biotech	China
TEMED	A610508-0100	BBI Life Science	China
Ammonium persulphate	A600072-0025	BBI Life Science	China
Chemiluminescence kit	WBKLS0500	Millipore	USA
TRIzol reagent	15596-026	Invitrogen	USA
StarScript II One-step RT-PCR Kit	A212-05	Genstar	China
2 × RealStar Green Power Mixture（with ROX II）	A314-10	Genstar	China
DAPI staining solution	C1006	Beyotime Biotechnology	China
Recombinant Mouse IL-23 Protein	1887-ML-010	R&D Systems	USA
Recombinant Mouse IL-1 beta/IL-1F2 Protein	401-ML-025	R&D Systems	USA
PMA	P-800-1mg	Sigma-Aldrich	USA
Ionomycin	I-700-1mg	Sigma-Aldrich	USA
Brefeldin A	B-275-1mg	Sigma-Aldrich	USA
Mouse IL-17A ELISA KIT	CME0041	4A BIOTECH	China
Mouse IL-22 ELISA KIT	CME0033	4A BIOTECH	China
Necrostatin-1 (Nec-1)	4311-88-0	Selleck Chem	USA
Z-VAD-FMK (Z-VAD(OMe)-FMK)	187389-52-2	Selleck Chem	USA
Fer-1	S7243	Selleck Chem	USA
RSL3	S8155	Selleck Chem	USA
FerroOrange	F374	DOJINDO	Japan
Adezmapimod (SB203580)	S1076	Selleck Chem	USA
U0126-EtOH	S1102	Selleck Chem	USA

Supplementary Table 2 . Antibodies used in this study

Name	Fluorochrome	Cat#	Vendor	Country
Biotin-anti-mouse CD4	-	100508	BioLegend	USA
Biotin-anti-mouse CD8a	-	100704	BioLegend	USA
Biotin-anti- mouse TER-119	-	116204	BioLegend	USA
Biotin-anti-mouse CD11c	-	117304	BioLegend	USA
Biotin-anti-mouse NK-1.1	-	108704	BioLegend	USA
Biotin-anti-mouse CD5	-	13-0051-85	eBioscience	USA
Biotin-anti-mouse TCR β chain	-	13-5961-85	eBioscience	USA
Biotin-anti-mouse TCR γ/δ	-	13-5711-85	eBioscience	USA
Biotin-anti-mouse CD3e	-	13-0861-86	eBioscience	USA
Biotin-anti-mouse Ly-6G	-	13-5931-86	eBioscience	USA
Biotin-anti-mouse CD11b	-	11-0112-86	eBioscience	USA
Biotin-anti-human/mouse CD45R/B220	-	103204	BioLegend	USA
Biotin-anti-human CD56 (NCAM)	-	13-0567-82	eBioscience	USA
anti-mouse NK1.1	PE-Cy7	25-5941-82	eBioscience	USA
anti- mouse CD45	APC-eFluor 780	47-0451-82	eBioscience	USA
STREPTAVIDIN	FITC	11-4317-87	eBioscience	USA
anti- mouse-CD90.2(Thy1.2)	PE-Cyanine7	25-0902-82	eBioscience	USA
anti-mouse CD127	PB	48-1271-82	eBioscience	USA
anti-mouse-CD4	AF700	56-0042-82	eBioscience	USA
anti-mouse RORγt	PerCP-eFluor710	46-6981-80	eBioscience	USA
anti -mouse CD196	APC	129814	BioLegend	USA
anti-mouse CD335(NKp46)	PE	12-3351-82	eBioscience	USA
anti-human/mouse Gata-3	PE	12-9966-42	eBioscience	USA
anti-mouse/human T-bet	eFluor660	50-5825-82	eBioscience	USA
anti-mouse Ly6G/Ly6C(Gr1）	PE-Cyanine7	25-5931-82	eBioscience	USA
anti mouse CD11b	FITC	11-0112-85	eBioscience	USA
anti-mouse IL-22	PE	12-7221-82	eBioscience	USA
anti-mouse/rat IL-17A	eflour660	50-7177-82	eBioscience	USA
Donkey anti-rabbit IgG(min,x-reactivity)	PE	406421	BioLegend	USA
NGAL(H-7)	-	sc-515876	santa	USA
ATF4(B-3)	-	SC-390063	santa	USA
xCT Antibody(SLC7A11)	-	DF12509	Affinity	USA
Gpx4(E-12)	AF647	sc-166570	santa	USA
Phospho-p38 MAPK (Thr180, Tyr182) Monoclonal Antibody (4NIT4KK)	PE	12-9078-42	eBioscience	USA
Phospho-ERK1/2 (Thr202, Tyr204) Monoclonal Antibody (MILAN8R)	PE	12-9109-42	eBioscience	USA

Supplementary Table 3. Sequences of primers used in this study

Primers name	Sequence	Primers name	Sequence
Ms-β-actin-for	5'-CGTGCGTGACATCAAAGAGAAG-3'	Ms-β-actin-rev	5'-CGTTGCCAATAGTGATGACCTG-3'
Ms-Gpx4-for	5’-GATGGAGCCCATTCCTGAACC-3’	Ms-Gpx4-rev	5’-CCCTGTACTTATCCAGGCAGA-3’
Ms-Acsl4-for	5’-CCTTTGGCTCATGTGCTGGAAC-3’	Ms-Acsl4-rev	5’-GCCATAAGTGTGGGTTTCAGTAC-3’
Ms-Reg3g -for	5’-CAGACAAGATGCTTCCCCGT-3’	Ms-Reg3g -rev	5’-GCAACTTCACCTTGCACCTG-3’
Ms-Reg3b -for	5’-CCCAGGCTTATGGCTCCTAC-3’	Ms-Reg3b-rev	5’-ATGGAGCCCAATCCAAGTGT-3’
Ms-Atf4-for	5’-ATGGCGCTCTTCACGAAATC-3’	Ms-Atf4-rev	5’-ACTGGTCGAAGGGGTCATCAA-3’
Ms-Slc7a11-for	5’-GGCACCGTCATCGGATCAG-3’	Ms-Slc7a11-rev	5’-CTCCACAGGCAGACCAGAAAA-3’
Ms-Slc3a2-for	5’-TGATGAATGCACCCTTGTACTTG-3’	Ms-Slc3a2-rev	5’-GCTCCCCAGTGAAAGTGGA-3’
Ms-Ptgs2-for	5’-AATACTGGAAGCCGAGCACCT-3’	Ms-Ptgs2-rev	5’-ACACCCCTTCACATTATTGCAGA-3’
Ms-Sat1-for	5’-GAGAACACCCCTTCTACCACT-3’	Ms-Sat1-rev	5’-GCCTCTGTAATCACTCATCACGA-3’
Ms-Hspb1-for	5’-ATCCCCTGAGGGCACACTTA-3’	Ms-Hspb1-rev	5’-GGAATGGTGATCTCCGCTGAC-3’
Ms-Nfe2l2-for	5’- TCTTGGAGTAAGTCGAGAAGTGT-3’	Ms-Nfe2l2-rev	5’-GTTGAAACTGAGCGAAAAAGGC-3’
Ms-Ets1-for	5’-AGTTTCAGCCATCACAACACA-3’	Ms-Ets1-rev	5’-GAAATCCTACCTGACGAGCAC-3’
