# Supplementary material for: GPX4 restricts ferroptosis of NKp46+ILC3s to control intestinal inflammation
Source: Cell Death Dis. 2024 Sep 19;15(9):687. doi: 10.1038/s41419-024-07060-3 (PMC11413021; doi:10.1038/s41419-024-07060-3)

Supplementary Material for

GPX4 restricts ferroptosis of NKp46^{+}ILC3s to control intestinal inflammation

Xinyao Li[1,2†], Junyu He[2†], Xiang Gao[3†], Guilang Zheng[4], Chunling Chen[4], Yimin Chen[2], Zhe Xing[2], Tianci Wang[2], Jian Tang[3], Yuxiong Guo[4*] & Yumei He[1,2*]

This file contains full and uncropped western blots:

The areas for the blots in the figures are indicated with a lined box.

Supplementary Figure 9 a.

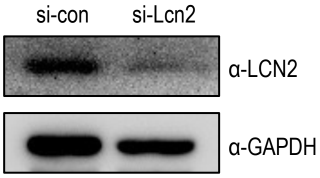

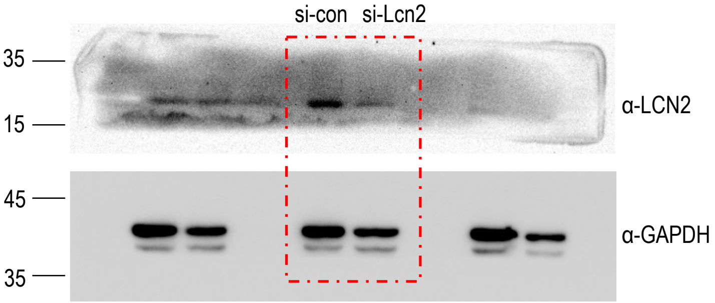

Supplementary Figure 9 e.

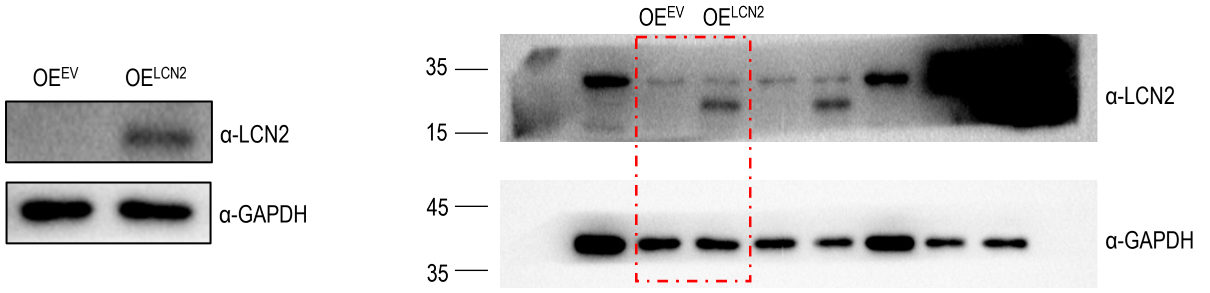

Supplement: Supplementary file 2 — Full and uncropped western blots [file 41419_2024_7060_MOESM2_ESM.pdf]
